# CURIE: Towards an Ontology and Enterprise Architecture of a CRM Conceptual Model

**DOI:** 10.1007/s12599-022-00744-0

**Published:** 2022-02-21

**Authors:** Miguel Fernández-Cejas, Carlos J. Pérez-González, José L. Roda-García, Marcos Colebrook

**Affiliations:** 1 Itop Management Consulting, Santa Cruz de Tenerife, Canary Islands Spain; 2 Depto. Matemáticas, Estadística e Investigación Operativa, Universidad de La Laguna (ULL), La Laguna (Tenerife), Spain; 3 Depto. Ingeniería Informática y de Sistemas, Universidad de La Laguna (ULL), La Laguna (Tenerife), Spain

**Keywords:** CRM conceptual model, Customer relationship management, Ontology, Enterprise architecture, Customer intelligence, Customer experience, Analytical CRM, Business analytics, Tourism

## Abstract

Companies face the challenge of managing customer relationships (CRM) in a context marked by a drastic digital transformation and unbridled evolution of consumer behavior, exacerbated by the COVID-19 pandemic. The customer is more demanding, has access to the global market and interacts with companies through multiple digital channels, such as email, social networks, mobile apps or instant messaging. In this situation, the success of a CRM implementation highly depends on information technology and the applications used. To harmonize this new business context with the development of information systems (IS), a suitable CRM ontology and enterprise architecture (EA) is needed. While an ontology-based conceptual model provides a unifying framework, aids sharing and reusing knowledge, and facilitates communication within a domain, an EA-based model unequivocally describes, analyzes, and visualizes how an organization should operate from the perspective of business, application, and technology. The purpose of the paper is the proposal of CURIE-O, a CRM OntoUML UFO-based ontology, together with CURIE-EA, a CRM ArchiMate-based EA to serve business managers and IS specialists an updated unifying framework of reference in the CRM domain as well as a highly efficient tool to support application development and maintenance in this changing and increasingly digital context. Modeling has proven to be an essential element to achieve high-performance information systems. In order to apply the ontology and the EA proposed here, the authors developed a CRM task management application prototype that was implemented as a case study in a consulting company. The methodology followed was design science research (DSR), in order to design and validate the artifacts. Within the DSR framework, other complementary research methods have been used, in particular literature research, interviews and focus groups carried out with several hotel chains in Tenerife (Canary Islands). The main existing CRM models in the scientific literature have also been analyzed together with the leading CRM market solutions.

**Supplementary Information:**

The online version contains supplementary material available at 10.1007/s12599-022-00744-0.

## Introduction

In his book *The Practice of Management*, Peter Drucker declares there is only one purpose of a business: to create a customer (Drucker 2007, 2018). Companies depend on their customers so they need to manage their relationships and try to keep them profitable and durable (Zaby and Wilde 2018). The main business processes in charge of these management tasks are marketing, sales and service.

Indeed, these processes have become much more complex since customer relationships have changed. A few years ago, customers still made most purchase decisions based on news, adverts and direct mail. The power of the internet and other technologies has completely revolutionized the way customers buy. They are much more informed and demanding and they have an increasing number of channels through which they can easily access a huge offering. If companies do not meet the needs of their customers, there is always a competitor just one click away.

On the other hand, customers have also started placing their experience above other factors such as brand reputation, price or just good service. They also buy following word-of-mouth recommendations, therefore companies must be sure they are not only satisfied but also brand advocates. Organizations must get to know their customers and find out what they need and want. They must continually evolve and adapt accordingly to meet increasing customer expectations. Those who are flexible and respond to these constant shifts will have a competitive advantage.

In order to handle this situation, CRM software emerged. CRM stands for ”Customer Relationship Management” and appeared in the information technology industry in the mid-1990s. The same term CRM is often used either in the context of technology or in business strategy. Payne and Frow (2005) suggested that CRM can be defined from at least three perspectives:

1. CRM is about the implementation of a specific technology solutions provider.
2. CRM is the implementation of an integrated series of customer-oriented technology solutions.
3. CRM is a holistic approach to managing customer relationships to create shareholder value.

In the current digital context, the success of a CRM implementation highly depends on information technology and how well CRM applications are adapted to company requirements (Widjaja and Budiardjo 2014). Research suggests that alignment between business strategies and IT increases profitability and the ability to gain a sustainable competitive advantage (Baker et al. 2011). To harmonize this new business context within the development of information systems (IS), a suitable model is needed.

In the scientific literature, there are several CRM conceptual models that have followed different strategies in their design: EA-based, ontology-based, and so on. Ontology-based models provide a unifying framework, help share and reuse knowledge, and facilitate communication within a domain. EA-based models unequivocally describe, analyze, and visualize how an organization should operate from a business, application, and technology perspective.

The purpose in this paper is:

- To review, reuse and reengineer CRM knowledge resources (ontological and non-ontological) to propose a CRM ontology, called CURIE-O (CUstomer Relationship, Intelligence and Experiences Ontology), based on the Unified Foundational Ontology (UFO) by using the ontologically well-founded language OntoUML.
- To design an ArchiMate-based enterprise architecture, called CURIE-EA (CUstomer Relationship, Intelligence and Experiences Enterprise Architecture) based on the CRM ontology proposed.
- To develop an application prototype based on both the ontology and the EA proposed in this paper, as well as implementing the prototype in a company as a case study in order to see the applicability of our models in a real-world case. For the prototype, we chose the CRM activities management process, for being transversal to the entire CRM process. For the case study, we chose to implement the prototype in a consulting services company, due to its proximity to the research team and because the customer relationships management in the service industry is especially important.

Among our motivations, we can mention the following:

- CRM is a key issue for a company, as without proper customer management, a company cannot survive.
- We understand that existing CRM conceptual models might need to be reviewed because of changes in customer relationships and customer behaviors in recent years, mainly due to digital transformation.
- An updated CRM ontology and EA in the current context could provide a useful tool for business managers and IT specialists to better understand the logic of CRM, to have a framework of reference in the CRM domain and to offer a highly efficient tool to support application development and maintenance.
- Modeling, in all its various forms, plays an important role in representing and supporting complex human design activities. Especially in IS analysis, as well as reengineering, modeling has proven to be an essential element to achieve high-performance information systems (Verdonck et al. 2019).
- Finally, there has not been much research done on ontologies in the CRM domain, and none based on UFO, one of the most used ontologies (Verdonck and Gailly 2016).

The methodology followed has been Design Science Research (DSR). This methodology establishes the steps to propose a solution to a problem through the design of artifacts, which is exactly our goal: a CRM conceptual model. Nowadays, DSR is an important and legitimate research focus in the IS field (Dresch et al. 2015). Within the DSR framework, other complementary research methods have been used, in particular literature research, interviews and focus groups carried out with several hotel chains in Tenerife (Canary Islands).

The main existing CRM conceptual models in the scientific literature have also been analyzed together with the leading CRM market solutions and other customer related aspects such as customer experience management, customer intelligence, quality management or changes in consumer habits post COVID-19.

**Table 1:** Main CRM conceptual models

Model	Main focuses
IDIC (Identify, differentiate, interact, customize) (Peppers and Rogers 2016a)	Process, information
QCi (Quality competitiveness index) (Feng and Jiadong 2008)	Strategy, process, organization, information, technology
Payne’s five-process (Payne 2012)	Strategy, process, information, technology
Gartner’s eight building blocks of CRM (Kinnett 2017)	Strategy, process, organization, information, technology
Buttle’s value chain (Buttle and Maklan 2019)	Strategy, process, organization, information, technology

The content of this article is divided as follows. After this introduction, in Sect. 2 we present a literature review on CRM conceptual models and we describe the theoretical background behind the CRM topic. In Sect. 3, we introduce the research methodology used for getting the insights needed for developing our model. A proposal of an ontology-based CRM model is shown in Sect. 4 whereas the EA-based CRM model is shown in Sect. 5. In order to apply the ontology and the EA to one of their possible use cases, in Sect. 6, we describe an application prototype for CRM task management that was later implemented in a consulting company as a case study. Section 7 presents a discussion of the implications and limitations of our CRM artifacts and, finally, the future lines of research and conclusions are presented in Sect. 8.

## Theoretical Background

### CRM Conceptual Models Review

Modeling plays an important role in representing and supporting complex human design activities. A conceptual model is a map of concepts and their relationships (Verdonck et al. 2019). It describes the key elements of a system, their characteristics and the associations among them. The aim of a conceptual model is to increase understanding and communication of a system or domain among stakeholders.

Based on related literature, there are several CRM conceptual models developed by different researchers. Table 1 shows a comparison of the main ones. These models follow different approaches, such as Pedron and Saccol (2009):

- *Philosophical*: CRM is understood as a way of doing business where the culture of the company is oriented towards customers and to building long-term relationships with them.
- *Strategic*: CRM is understood as the way in which the company will succeed in building and managing relationships with customers, establishing lines, objectives and actions.
- *Process-oriented*: CRM is understood as a business process responsible for managing customer relationships, and as such, it will have its objectives, procedures and managers.
- *Organizational*: CRM is understood as a set of tasks to be performed by different roles and departments.
- *Technological*: CRM is understood as a tool that enables the management of customer relationships.

Although the technological component is a common part of most of those models, they do not go far enough. Without proper information technology, a successful and good CRM implementation will be very difficult to obtain (Widjaja and Budiardjo 2014). This is due to the complexity of managing customer relationships in a context characterized by omnichannels, and the speed and volume of information to be treated. For this reason, our proposed CRM conceptual model has a special focus on technology and analytics.

### CRM Main Concepts

#### Customer Relationship Stages

**Fig. 1:**
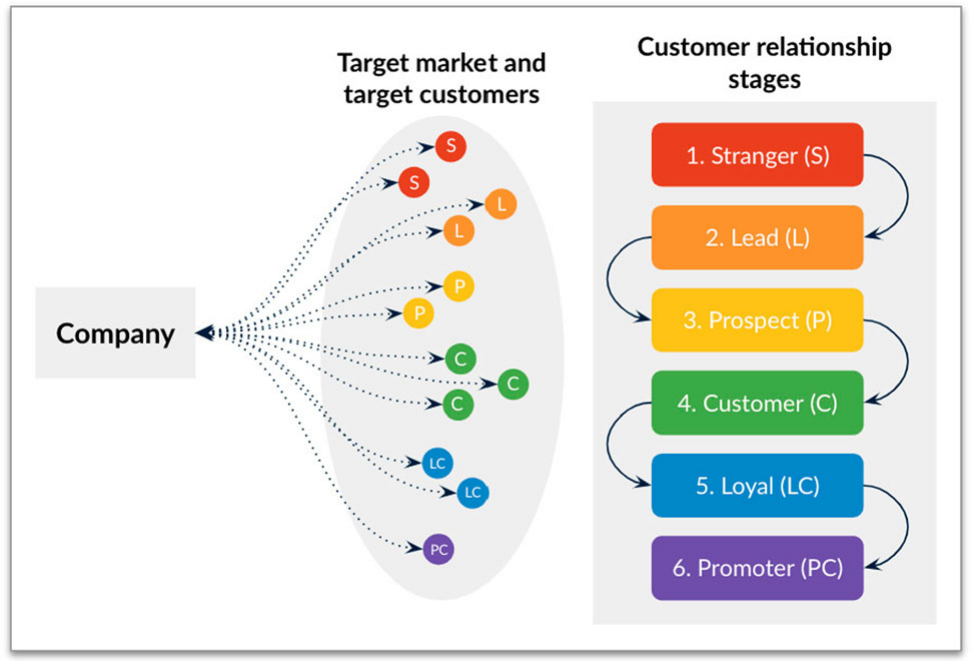
Customer relationship stages from the company’s perspective

As we can see in Fig. 1, a company has a target market where their customers live. These customers can be at various stages depending on the extent of their relationship with a company (Chouaib Dakouan and Redouane Benabdelouahed 2019):

1. *Stranger*: When the company does not have any customer data.
2. *Lead*: When the company has customer data enough to have a conversation with him/her.
3. *Prospect*: When the customer has shown some interest in products or services.
4. *Customer*: When the first purchase has taken place.
5. *Loyal*: When the customer consistently purchases over an extended period of time.
6. *Promoter*: When the customer promotes the company, product or service.

If, as we indicated above, the purpose of a company is to create a customer (Drucker 2007, 2018), this means that organizations have to focus on taking their clients from the Stranger stage to the customer Promoter stage.

#### Buyer Persona

Depending on a company’s business, a customer might be a consumer or another organization. In any case, the relationship ends up being with people. This target customer is the *Buyer Persona*. Buyer persona is a customer profile, a semi-fictional representation of your ideal client, which is based on truthful information and informed guesses about demographics, behavior models, motivations and customer goals (Kelly et al. 2017).

A Buyer Persona helps organizations to identify where their ideal customers spend time so they can be present in those places. They also help to develop products and services, create appropriate content and messages and address specific problems and beliefs. Some of the results shown by companies that have developed and deployed their Buyer Persona are: (1) improvement in email opening rates and the business generated with them (2) much more effective and user-friendly websites and (3) increase in potential customers.

In order to create a Buyer Persona, several aspects must be taken into account as the common behavior models, common professional and personal problems, general objectives, wishes and dreams, as well as general demographic and biographical information.

#### Customer Journey and Customer Experience

The stages a customer follows in a purchase process are often referred to as a customer life cycle or customer journey. The detail of what happens on that trip depends on each client, but usually they are analysed in groups of profiles with similar behaviors, or, that is, Buyer Persona.

**Fig. 2:**
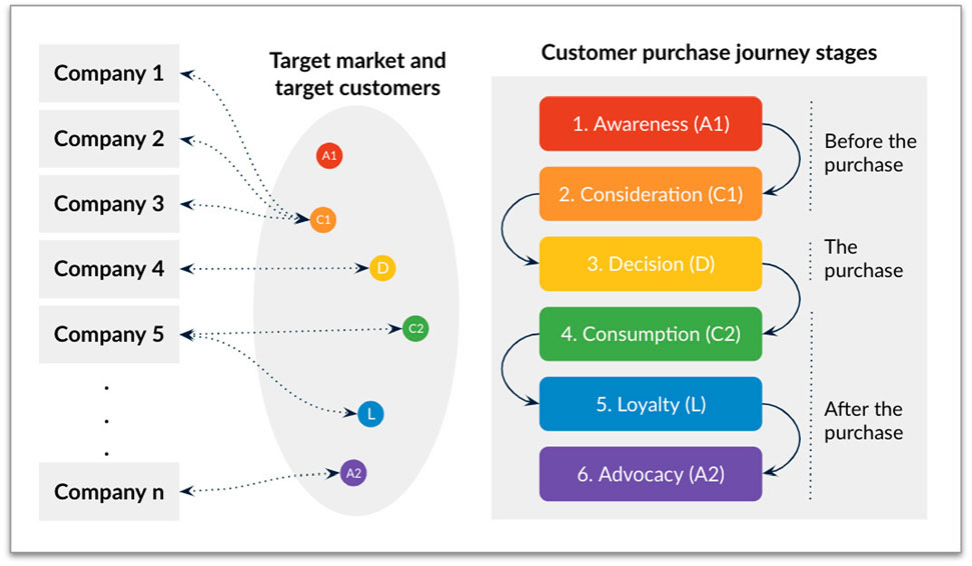
Customer journey

From a general point of view, this journey has different stages (Vázquez et al. 2014). As we can see in Fig. 2, in this paper we adopt the following widely agreed purchase stages:

1. *Awareness*: customers identify their problems or needs and decide whether or not they should be a priority.
2. *Consideration*: customers have clearly defined their problems or needs and have committed to addressing them. They explore different options and also express their preference towards a specific brand.
3. *Decision*: customers have already decided on a solution that best meets their needs, so they purchase that product/service.
4. *Consumption*: customers have purchased the product/service and start consuming or using it. The consumer actually becomes a customer of the brand and heavily interacts with the brand and gains much experience.
5. *Loyalty*: customers consider retaining their relationship with the same brand or moving to a different provider, based on the level of satisfaction and resultant behavior after purchase and consumption.
6. *Advocacy*: customers are satisfied with the brand, and are now providing referrals for its products or services.

Through this journey, companies and customers have many interactions or touch points where the customer experience takes place. There are different definitions of customer experience (abbreviated as CE or CX), but they all conclude that it is the product of a client’s perceptions after interacting rationally, physically, socially, sensually, emotionally and/or psychologically with any part of a company throughout its customer journey (Lemon and Verhoef 2016).

The business process in charge of these issues is known as Customer Experience Management (CEM). Nowadays, this is a competitive differentiation strategy that goes beyond customer satisfaction and quality of service. It is about measuring, understanding, designing and managing customer interactions to meet or exceed their expectations and, therefore, increase their satisfaction and loyalty (Lemon and Verhoef 2016).

An important aspect of the touch points are the channels through which they take place. The number of channels used by the customer in the interaction with companies is increasing. Many of these channels are digital, such as email, social networks, chat or web. This situation forces companies to establish coherent and consistent communication and interaction through them in order to achieve the seamless experience demanded by the customer. This concept is known as Omnichannel Management (Juaneda-Ayensa et al. 2016). This communication has to follow a prescriptive approach that removes obstacles and guides customers through decision making, teaching them not only about what to buy but also on how to do it (Toman et al. 2017).

Therefore, it is key to understand customers’ purchase journeys, identify customer challenges at each buying stage, design the tools to help overcome each of those challenges and track customers’ purchase progress.

#### Communication Channels, Unstructured Data and Processing

The current number of communication channels that organizations use in their relationship with customers has increased considerably. Most of these channels share three characteristics: (1) they are digital (2) they are social and (3) they are mobile (Williams 2014). This situation complicates omnichannel management but, at the same time, offers new options to interact with customers, analyze their behavior, improve their segmentation and enhance the customer experience. Many of these digital channels that customers use during their purchase journey manage unstructured data. This means that a standardized format that could be easily processed by applications is not followed. Examples of this type of information are emails, text messages, documents, images or videos. Gartner estimates that 80% of an enterprise’s data is unstructured (Dayley and Logan 2015).

In order to extract insights effectively from this type of information, there are several issues that make this task difficult: the great variety of systems located inside and outside the company, manual data processing or the need for expert knowledge.

The good news is that this is now changing, thanks to new technologies such as: (1) Text Analytics, which automates the process of analyzing unstructured data to enable better understanding, and (2) Natural Language Processing (NLP), which applies semantic analysis to understand the context of the message, and the sentiment within it.

#### CRM Process, Funnel and Flywheel

**Fig. 3:**
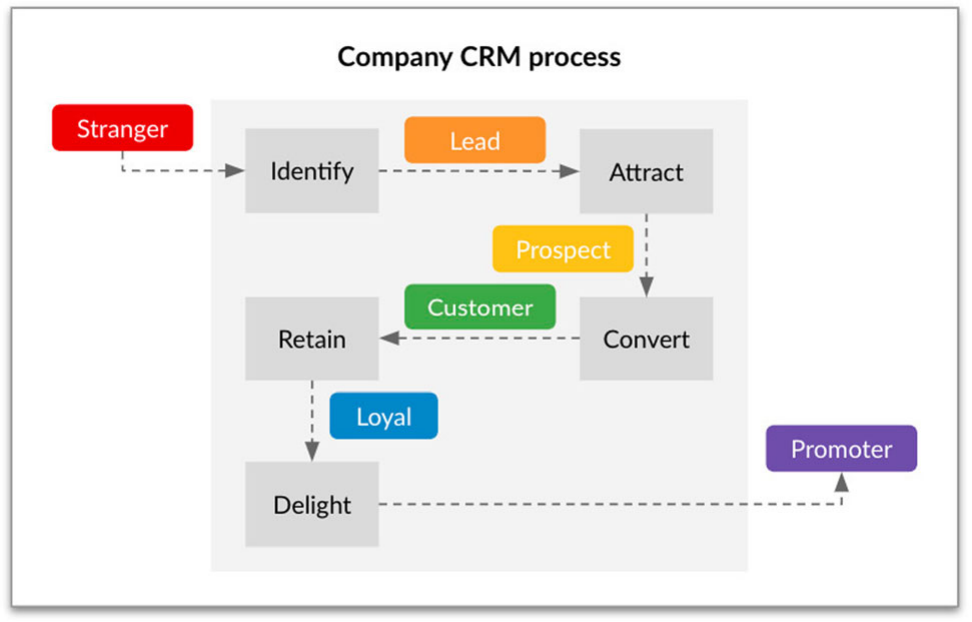
Company CRM process

Just as the customer follows his customer journey during the purchase process, a company follows a CRM process that seeks to take potential customers from the Stranger stage to the Promoter stage (Fig. 3). In most companies, this process consists of the following steps (Ngai et al. 2009):

1. *Identify*: Establish potential customer segments and gather some information so that the Stranger becomes a Lead.
2. *Attract*: Show the Leads they can trust you to help them solve their problems and needs so that they become a Prospect.
3. *Convert*: Have conversations with Prospects on the channels they prefer, capture detailed information about their needs, show your solutions and make quotes so that the Prospect becomes a Customer.
4. *Retain*: This regards customer satisfaction and up/cross selling so that the Customer becomes a Loyal.
5. *Delight*: This concerns exceeding a customer’s expectations to create a positive and memorable customer experience, converting them into a promoter and a reference for the brand.

It is important to realize that in order to improve the efficiency of this process, the company should know the precise purchasing process stage where the potential customer is. This helps in adapting messages and content to better communicate with the customer.

There are companies that simplify this process in three major subprocesses: marketing, sales and services. Identify and Attract subprocesses are usually executed by the marketing team, while Convert is responsibility of the sales team, and Retain and Delight are carried out by the customer services team.

**Fig. 4:**
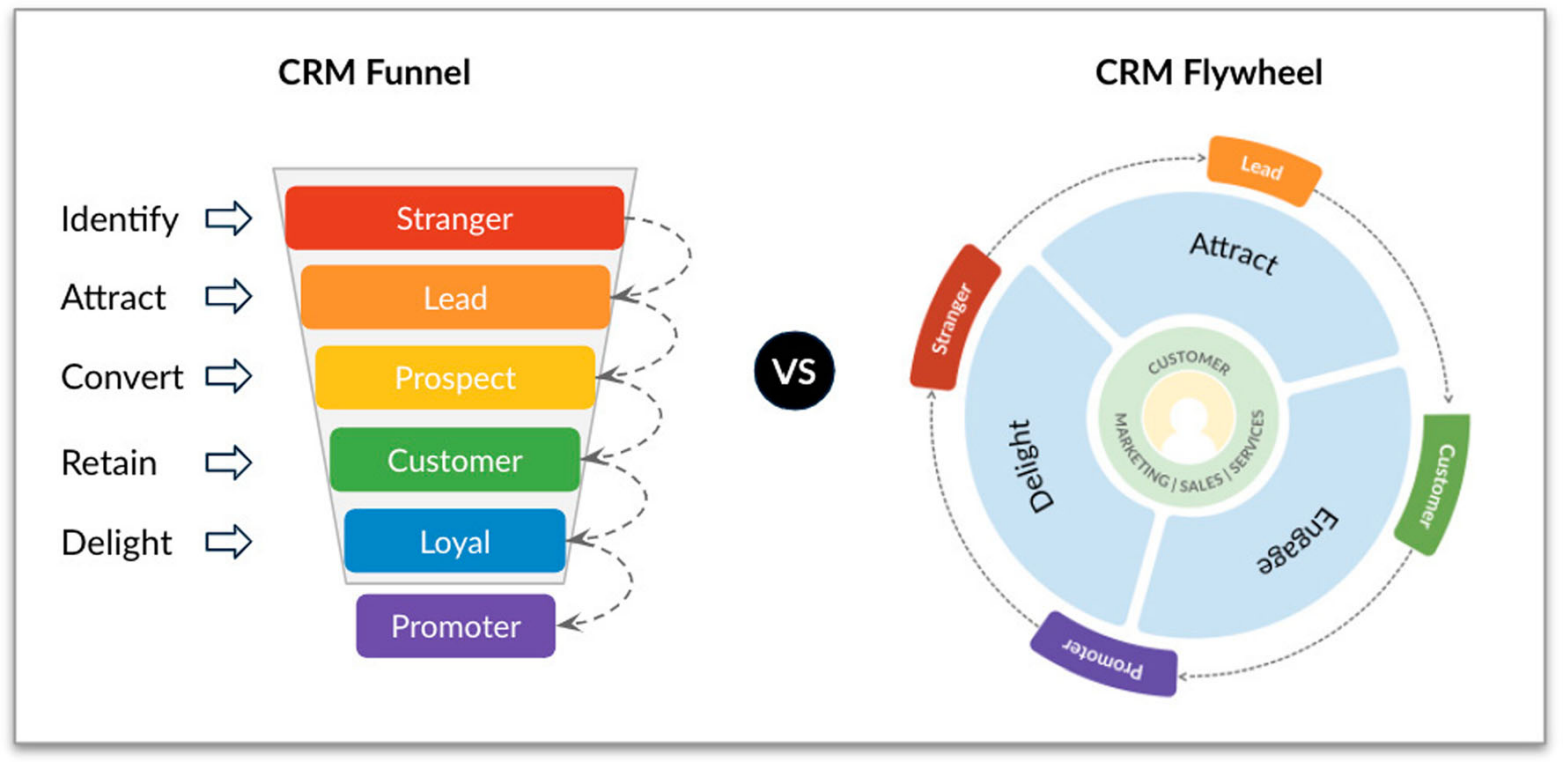
CRM funnel and flywheel

Like any other business process, the CRM process should have associated its Key Process Indicators (KPIs) as well as its visual graphs in order to track their performance and goals. The traditional way to show this information is through the CRM Funnel (Colicev et al. 2019), sometimes called Sales Funnel. This visual model has the shape of a funnel cut from top to bottom in as many sections as stages as the process has. Each section contains customers, whose status is that associated with the section, for example, the Identify section manages the customers in the Stranger status (Fig. 4).

However, the funnel model looks like the company’s objective is continuously attracting new customers (Halligan 2018). This may not be regarded as a problem, but if we look at some recent statistics on customer behavior (Kumar and Reinartz 2018) people increasingly engage in word-of-mouth on different social media platforms with friends, acquaintances but also strangers.

In this context, it is especially important that customers become promoters of the brand so they generate word-of-mouth and key recommendations for the success of the sales process. We have observed that a traditional CRM funnel does not visualize this need nor the management of it. According to Halligan (2018), the funnel representation can be replaced by a visual model based on a flywheel (Fig. 4) whose main characteristics are: (1) the customer is always in sight and located in the center, all sale process steps are equally important and the process is continuous, so it does not stop once you obtain a customer and (2) all teams have to take action in all the steps. The importance of improving the customer experience through to the delight is highlighted, which will produce satisfied customers and promoters of the brand.

### CRM Software, Big Data, and Machine Learning

Considering CRM as a customer-oriented technology solution, there are three differents types of CRM software, depending on its functionality (Croxatto 2005):

- *Collaborative CRM*: This is related to omnichannel. It offers the necessary tools for interaction, through different communication channels, between company and customers, showing a single business face. It covers both traditional channels (points of sale, offices, telephone) and digital channels (web, app, social networks, email, chat, etc.). This communication ensures that when a customer decides to use different channels, they will perceive the same final result.
- *Transactional CRM*: This is the best known and has been established in a large number of companies. It is used to record everything that customers request and receive from the company, relying on the different modules: marketing (leads, campaigns, etc.), sales (opportunities, contacts, offers, etc.) and customer service (tickets, support contracts, FAQ, etc.).
- *Analytical CRM*: This is concerned with gathering information about customer behavior and interaction, so companies can predict customer trends and suggest products with the goal of increasing customer satisfaction and customer retention rate.

The goal of analytical CRM is to provide relevant knowledge to guide and continually optimize the transactional CRM processes in terms of a closed loop architecture. In particular, integrating analytical CRM knowledge into decisions within the transactional CRM processes contributes to intelligent CRM (Zaby and Wilde 2018).

The technologies behind Analytical CRM are mainly Big Data and Machine Learning. They are a set of methodologies and technological tools developed for dealing with large and complex volumes of data and for learning from them, identifying patterns and helping to make better decisions.

Although many Machine Learning algorithms have been around for a while, the ability to automatically apply complex mathematical calculations to Big Data – again and again, faster and faster – is a recent achievement. There are three main Machine Learning methods (Vinothini and Baghavathi Priya 2017): supervised learning, unsupervised learning and reinforcement learning.

Data can be studied with a set of data analytics tools that provide varying amounts of value to an organization (Krumeich et al. 2016):

- *Descriptive*: This helps to answer the question ”What is happening”. It is the most widespread type of analysis and provides the usual management indicators.
- *Diagnostic*: This examines data or content to answer the question ”Why did it happen?” It is characterized by techniques such as drill-down, data discovery, data mining and correlations.
- *Predictive*: This helps to answer the question ”What is likely to happen”. Through the development of models based on historical data, it seeks to predict the likelihood of an event to happen.
- *Prescriptive*: This helps to answer the question ”What do I need to do”. From the data provided by the other types of analysis, advice on the best action to take.

In Ngai et al. (2009), the main types of data mining models used in analytical CRM are identified: Association, Classification, Clustering, Forecasting, Regression, Sequence Discovery and Visualization. There are numerous Machine Learning algorithms available for each type of those models. Selecting the right one should be based on the data characteristics and business requirements. Examples of these algorithms include: Decision Trees, Neural Networks, K-Nearest Neighbors, Linear Regression or Apriori.

As we discussed above, analytical CRM refers to the analysis of customer characteristics and behaviours so as to support customer relationship management strategies of the organization. Big Data and Machine Learning are then the right tools for analyzing customer’s data in a CRM. They allow the discovery of valuable information hidden in the data, transforming it into valuable and useful knowledge that helps companies make better decisions.

### Ontologies, UFO and OntoUML

Conceptual models were introduced to increase understanding and communication of a system or domain among stakeholders. Some commonly used conceptual modeling techniques and methods include: Business Process Model and Notation (BPMN), Entity Relationship Modeling (ER), Object-Role Modeling (ORM), and the Unified Modeling Language (UML). Many of these conceptual modeling techniques lacked an adequate specification of the semantics of the terminology of the underlying models, leading to inconsistent interpretations and uses of knowledge. In order to overcome such issues, ontologies can be applied. Ontologies can be described as a set of things whose existence is acknowledged by a particular theory or system of thought. Research on ontologies has become increasingly widespread and they can be applied to articulate and formalize the conceptual modeling grammar needed to describe the structure and behavior of the modeled domain (Verdonck et al. 2019).

Since the 1980s, there has been a growing interest in the use of foundational ontologies for evaluating and reengineering modeling languages and methodologies. A foundational ontology defines a system of domain-independent categories and their ties, which can be used to articulate the conceptualizations of reality. The use of foundational ontologies aims to ensure ontological correctness of the language and of the models described with the language (Guizzardi et al. 2015). One of the main foundational ontologies used by the scientific community is the Unified Foundational Ontology (UFO) (Verdonck and Gailly 2016).

Due to its coverage and level of expressiveness regarding philosophical meta-properties, the notions of material relations and relational properties (Ruy et al. 2014) as well as its widespread use among the scientific community, we have adopted the UFO foundational ontology in the present work.

OntoUML was conceived as an ontologically well-founded version of the UML 2.0 fragment of class diagrams targeted at human users. The language is based on the cognitive science achievements of understanding specifics of our perception and on modal logic and related mathematical foundations (sets and relations). Unlike other extensions of UML, OntoUML does not build on the UML’s ontologically vague ”class” notion, rather it constitutes a complete system, independent of the original UML elements. OntoUML is designed to comply with UFO and because of that, it provides expressive and precise constructs for modellers to capture a domain of interest. OntoUML addresses many problems in conceptual modelling and has been successfully applied in different contexts (Pergl et al. 2013).

An option for the implementation of a computational ontology is to use the light version of UFO, gUFO, that sacrifices some conceptual modeling ability to make it OWL (Ontology Web Language) compliant (Almeida et al. 2019). gUFO reflects UFO taxonomies of individuals and types (universals). By using gUFO, we are taking advantage of its semantics, rules, properties of predefined objects, conceptual patterns, different ways of representing qualities, to manage situations that change in time or automatic error detection. These features make it possible to obtain a domain-specific ontology faster and more consistently. gUFO leverages benefits that were only available to OntoUML users and to Semantic Web implementers. In addition, gUFO gets better integration between the taxonomy of types and taxonomy of individuals than in OntoUML (due to limitations of UML).

### Enterprise Architectures, TOGAF, and ArchiMate

With software applications becoming larger and sharing information among different business processes, the development of IT should be done in conjunction with the development of the context in which it is used. When the IT industry got confronted with complex structures and decisions, the idea of architecture was introduced as a means to align business and IT, enabling strategic planning, to assess risks, and to check compliance with legal regulations. Enterprise architecture (EA) supports the analysis and design of business-oriented systems through the creation of complementary perspectives from multiple viewpoints over the business, information systems and technological infrastructure, enabling communication between stakeholders (Op’t Land et al. 2009).

In order to support the design, modeling and management of the different components of an enterprise and its interaction, several enterprise architecture frameworks were proposed such as The Open Group Architecture Framework (TOGAF) (The Open Group 2018), the Zachman Framework (Zachman 1987) or The Department of Defense Architecture Framework (DoDAF) (U.S. Department of Defense 2010). Those frameworks typically divide the metamodel into three abstraction layers (Abdallah et al. 2019): Business, Application and Technology.

According to Abdallah et al. (2019), TOGAF is rated higher compared to other frameworks. ArchiMate is a standard modeling language for describing EA and its structure corresponds to TOGAF layers. It aims to support enterprise architects in describing, analyzing and visualizing the relationships among business domains in an unambiguous way.

## Research Methodology

This paper follows Design Science Research (DSR) methodology. DSR is now an important and legitimate research approach in several fields, including IS field (Dresch et al. 2015). According to March and Smith (1995), this methodology establishes the steps to propose a solution to a problem through the design of innovative artifacts such as:

- Constructs provide the language in which problems and solutions are defined and communicated.
- Models are a set of propositions or statements expressing relationships among constructs. They represent situations as problem and solution statements.
- Methods define processes. A Method is a set of steps (an algorithm or guideline) used to perform a task. They are based on a set of underlying constructs (language) and a representation (model) of the solution space.
- Instantiations show that constructs, models, or methods can be implemented in a working system. They demonstrate feasibility, enabling concrete assessment of an artifact’s suitability to its intended purpose.

**Table 2:** Applied methodologies

Research activities	Research outputs			
	Models			Instantiations
	CRM Ontology	CRM Enterprise Architecture		Task Manager prototype
Identify problem and motivate	Literature review	Literature review		Literature review
Define objectives of a solution	Literature review	Literature review		Literature review
Design and development	Literature review	Literature review		Conceptual research
	Interviews			
	Focus groups			
Demonstration	Instantiations	Instantiations		Case study
Evaluation	Instantiations	Instantiations		Case study
Communication	To be published in an academic journal	To be published in an academic journal		To be published in an academic journal

The research steps or activities proposed by DSR vary slightly depending on the author. Peffers et al. (2007) looked to influential prior research and proposed a DSR process consisting of six activities:

1. *Identify problem and motivate*: Define the specific research problem and justify the value of a solution. Since the problem definition will be used to develop an artifact that can effectively provide a solution, it may be useful to atomize the problem conceptually so that the solution can capture its complexity. Justifying the value of a solution accomplishes two things: it motivates the researcher and the audience of the research to pursue the solution and to accept the results, so it helps to understand the reasoning associated with the researcher’s understanding of the problem. Resources required for this activity include knowledge of the state of the problem and the importance of its solution.
2. *Define objectives of a solution*: Infer the objectives of a solution from the problem definition and knowledge of what is possible and feasible. The objectives can be quantitative, e.g., terms in which a desirable solution would be better than current ones, or qualitative, e.g., a description of how a new artifact is expected to support solutions to problems not addressed until now. The objectives should be inferred rationally from the problem specification. Resources required for this include knowledge of the state of problems and current solutions, if any, and their efficacy.
3. *Design and development*: Creation of the artifact. Conceptually, a design research artifact can be any designed object in which a research contribution is embedded in the design. This activity includes determining the artifact’s desired functionality and its architecture and then creating the actual artifact. Resources required moving from objectives to design and development include knowledge of theory that can be brought to bear in a solution.
4. *Demonstration*: To demonstrate the use of the artifact to solve one or more instances of the problem. This could involve its use in experimentation, simulation, case study, proof, or other appropriate activity. Resources required for the demonstration include effective knowledge of how to use the artifact to solve the problem.
5. *Evaluation*: Observation and measurement of how well the artifact supports a solution to the problem. This activity involves comparing the objectives of a solution to actual observed results from use of the artifact in the demonstration. It requires knowledge of relevant metrics and analysis techniques. Depending on the nature of the problem venue and the artifact, evaluation could take many forms. It could include such items as a comparison of the artifact’s functionality with the solution objectives from activity two above, objective quantitative performance measures, such as budgets or items produced, the results of satisfaction surveys, client feedback, or simulations. It could also include quantifiable measures of system performance, such as response time or availability. Conceptually, such evaluation could include any appropriate empirical evidence or logical proof.
6. *Communication*: Communicate the problem and its importance, the artifact, its utility and novelty, the rigor of its design, and its effectiveness to researchers and other relevant audiences. In research publications, researchers might use the schema of this process to structure the paper.

Our study has a research output of several artifacts proposed to satisfy stakeholders’ (business managers and IT specialists) goals in a given context (CRM). Thus, we decided to adopt DSR methodology as our research strategy and follow this six-step process. We cover all the research activities and have a research output of two models and one instantiation:

- The first research goal is to build an ontology (i.e. artifact or model) that makes it possible to conceptually express the CRM logic in a structured form. In Sect. 4.1 we identify the problem and motivation as well as the objectives of the solution. In Sect. 4.2 we show the steps followed for the development of this artifact and in Sect. 4.3 we evaluate the ontology.
- The second research goal is to design, based on that ontology, an CRM enterprise architecture. In Sect. 5.1 we identify the problem and motivation as well as the objectives of the solution. In Sect. 5.2 we show the steps followed for the development of this artifact as well as the results obtained, and in Sect. 5.3 we evaluate the EA model.
- The third research goal consists in applying the ontology and the EA to one of its possible uses (i.e. instantiation). We chose the instantiation of the part of the ontology related to the CRM activities in an IT application developed under the proposed enterprise architecture. In Sect. 6.1 we identify the problem and motivation as well as the objectives of the solution. In Sect. 6.2 we show the application prototype and in Sect. 6.3 we evaluate the application with a case study of implementation in a consulting company.

The sixth step (communication) of the three artifacts is implicit in this paper. To achieve these specific research objectives, the DSR methodology contemplates the use of other methodologies (March and Smith 1995). Some of these complementary methodologies are: speculation / commentary, literature review, literature analysis, case study, interview and secondary data. According to each of our objectives, we have chosen the methodologies shown in Table 2.

## Artifact 1: Ontology-Based CRM Model

### Problem, Motivation, and Objectives of the Solution

#### Problem and Motivation

According to Zaby and Wilde (2018), the internet has made customers better informed, more networked and flexible, and thus, more powerful than ever before. Their demands are constantly growing and changing. At the same time, competitors are also better informed and more flexible, and competitive pressure in the market is increasing. Consequently, well-working customer relationship management (CRM) is becoming a critical source of competitive advantage.

The first step to efficiently manage the relationship with clients is to know in detail CRM scope and what are the concepts and relationships behind it, that is, have a conceptual model of CRM. This knowledge could be useful for business managers by helping them better understand what the key elements of customer management are and establish a common language for communication within the company. It would also allow IT specialists to be clear about the entities and relationships that they should take into account when developing CRM applications. Modeling has proven to be an essential element to achieve high-performance information systems (Verdonck et al. 2019).

Although there are research works in this line, the literature has not yet examined this topic in detail. Furthermore, understanding that CRM has been affected due to changes in customer behavior and digital transformation, motivates us to investigate and propose a CRM model according to this new context.

We consider the problem of CRM modeling relevant as it is something common to every company and clearly affects its competitiveness. It is also a complex but finite problem since the concepts, actors, processes and technologies involved can be identified and studied in a relatively manageable time.

To know the current context of the problem, we investigate the state of the art of CRM by reviewing the literature. The summary of the information obtained is shown in Sect. 2.

#### Objectives of the Solution

Our solution proposal for the problem of modeling a CRM is the development of a domain ontology that will allow to conceptually express the CRM logic in a structured form. As we pointed out in Sect. 2.4, an ontology is an explicit specification of a conceptualization that includes definitions of concepts and their interrelations. The ontology is used to improve communication between humans by classifying vocabulary and taxonomy that models a domain through objects, concepts, properties and relations. We selected the ontology conceptual modeling technique because it offers an adequate specification of the semantics of the terminology leading to consistent interpretations and uses of knowledge.

The objectives of our solution are mainly:

- Propose an updated ontological conceptual model that includes the concepts and relations of CRM in the new digital context. This will allow the actors of our solution to have a more realistic ontology with the current situation.
- Propose a CRM ontology based on the foundational ontology UFO. There are currently no CRM ontologies based on UFO, even though it is one of the most used in the scientific literature due to its coverage and level of expressiveness, as well as offering its own modeling language, OntoUML.

### Design and Development

As mentioned above, our objective is the design and development of a CRM ontology adapted to the current context by using the foundational UFO ontology. An ontology can be developed from scratch or from reusing other ontologies. Reusing ontologies and reengineering them has the advantage of reducing the necessary work and increasing the quality of the ontologies obtained by reusing components already tested. When choosing these ontologies, it is also recommended to take a foundational ontology as a reference. These ontologies define a system of domain-independent categories and their ties, which can be used to articulate the conceptualizations of reality. The use of foundational ontologies aims to ensure ontological correctness of the language and of the models described with the language.

We have decided to reuse existing knowledge resources on CRM. The methodology we followed is NeOn (Suárez-Figueroa et al. 2015) that gives us general guidelines for knowledge resources searching and selecting, as well as reusing ontology and non-ontology resources and applying reengineering. The NeOn methodology is based on nine scenarios for building ontologies and ontology networks. Our work will be mainly based on scenario 4 (reusing and reengineering ontological resources) and scenario 2 (reusing and reengineering non-ontological resources). The methodology proposes a set of activities that, in general, are: (1) specification of ontology requirements, (2) reuse of non-ontological resources, (3) reengineering of non-ontological resources, (4) reuse of ontological resources, (5) reengineering of ontological resource.

#### Specification of Ontology Requirements

In this activity, we defined the set of requirements that the ontology should satisfy after being formally implemented. Most of the existing methodologies suggest the identification of competency questions (CQs) as the technique for establishing the ontology requirements (Suárez-Figueroa et al. 2015).

CQs are questions written in natural language that ontology should be able to answer. This initial set of ontology requirements is obtained by means of a set of interviews with users and domain experts. We chose the following CQs:

- *CQ1*: What are the tasks to be carried out by the company based on the relationship status of a customer?
- *CQ2*: What are the tasks that a customer performs based on the status of the customer journey they are in?
- *CQ3*: What are the tasks of a specific channel?
- *CQ4*: What are the stages of the customer journey?
- *CQ5*: What are the customer relationship stages?
- *CQ6*: What are the tasks to be carried out by the company depending on the department to which you belong?

**Fig. 5:**
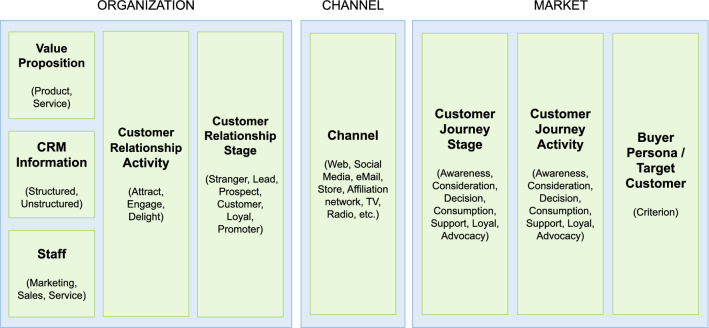
The nine CRM model building blocks

Another kind of requirement that we should take into account when building a domain ontology is the content requirement derived from the domain analysis which provides the knowledge engineer with the concepts and relations that the ontology should model. From our domain analysis, we identified nine main building blocks that constitute the essential CRM model. These elements, presented in Fig. 5, are a synthesis of the CRM model literature review described in Sect. 2 and consist of Value Proposition, Information, Staff, Customer Relationship Activity, Customer Relationship Stage, Channel, Customer Journey Stage, Customer Journey Activity and Buyer Persona.

Finally, we also decide to base the proposed ontology on a foundational ontology as another requirement, providing a rich characterization of the basic concepts and relations on which specific CRM concepts and relations can be formally defined. The reuse of existing ontological models, and especially upper level ontologies, is of major importance in semantic knowledge representation, mainly because: (1) linking a domain ontology to an existing well-grounded upper level model guarantees a higher degree of accuracy in the definition of basic concepts; (2) making reference to the same upper level ontology enhances the interoperability between different domain ontologies; and (3) in domains characterized by high heterogeneity of concepts, upper level ontologies provide a coherent framework supporting the management of such heterogeneity.

**Fig. 6:**
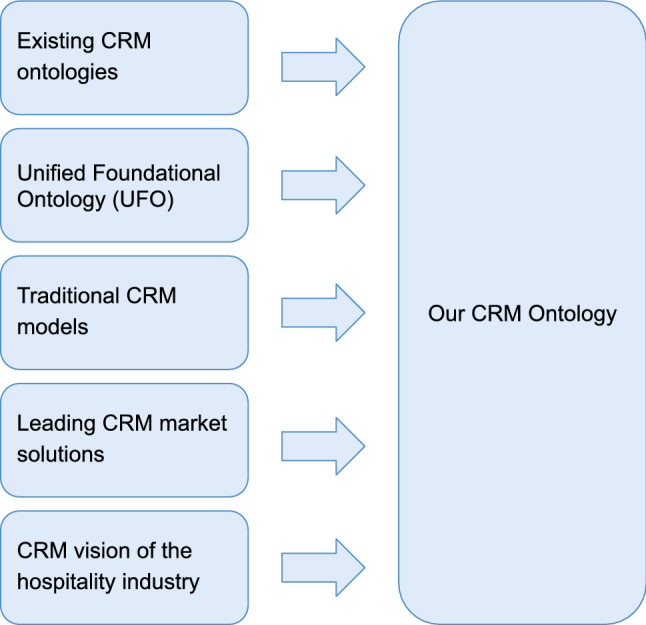
Knowledge sources used for our proposed CRM ontology

We reviewed existing CRM ontologies, the UFO foundational ontology, the non-ontology based CRM conceptual models, the features of the main market CRMs and the specific vision of CRM in the hospitality sector (see Fig. 6).

#### Reuse of Non-Ontological Resources

Applying scenario 2 of the NeOn methodology (reusing and reengineering non-ontological resources), we searched for non-ontological CRM sources of knowledge in the following different ways:

- *Scientific literature for traditional CRM models*: The details of analyzed and selected models are shown in Sect. 2.1. They were: IDIC (Identify, Differentiate, Interact, Customize) (Peppers and Rogers 2016b), QCI (Feng and Jiadong 2008), Payne’s Five-Process (Payne 2012), Gartner’s Eight Building Blocks of CRM (Kinnett 2017) and Buttle’s Value Chain (Buttle and Maklan 2019).
- *Current market leading CRM application*s: We identified the main common features that developers are offering. We analyzed: (1) The CRM solutions selected by Cruz and Vasconcelos (2015) (SugarCRM, Salesforce, Oracle, Microsoft Dynamics CRM and Sage CRM) where they reviewed their functional characteristics and make a final selection of those that are common to all of them; (2) One of the CRM software comparison reports offered by market research companies that shows a fairly exhaustive comparison of 40 different CRM applications (Business-Software 2021). This report presents a summary of the functional characteristics that each of these applications has. Without getting into issues of the rigor of this comparison, the knowledge it contributes on the features of these applications seems interesting to us. The reports contain 175 features that we have merged and grouped, obtaining 44 main concepts; (3) Gartner Magic Quadrants reports about the most demanded functionalities for CRM applications (Gartner, Inc. 2021). In particular, we reviewed: CRM Lead Management, Sales Force Automation, Multichannel Marketing Hubs and Voice of the Customer.
- *Hospitality industry*: We contacted a group of hotels, a sector with a clear orientation towards customer management, in order to get insights about their CRM process. We conducted: (1) research interviews by organizing one-on-one meetings and using both open and closed questions to gather quantitative data and also giving the interviewee the freedom to express themselves. We wanted to get a deeper understanding of a user’s point of view regarding CRM requirements. The interviews were organized at seven local hotel groups at which the questionnaire was conducted. 60% were hotels belonging to a chain, while 40% were private. 63% were four-star hotels, 25% five-star hotels and 12% aparthotels. All were located in Tenerife, Canary Islands, and fit into the category of sun-and-beach hotels. The people interviewed were hotel managers and marketing/sales directors. From these hotel interviews we obtained a variety of results. The most valued CRM attributes were its agility, its ability to integrate with the PMS (hotel management system), the option to be acquired with the necessary modules, its analytical capacity and its versatility to meet the custom requirements of each business. Regarding marketing features, more than 90% of the interviewees indicated the management of the Buyer Persona, the campaigns and the management of the marketing plan as the most important issues. As for the sales process, the sales plan, accounts and contact management as well as cross-selling or online sales were the most valued issues. And finally, regarding service process, they highlighted customer satisfaction management, customer web interaction and project management; (2) focus group sought to facilitate a discussion about the CRM process in an real market environment. In this work session, 15 people participated from five different hotel groups in Tenerife. Their roles were hotel manager, marketing director, IT director and technical director. The techniques used in the session were Design Thinking and Brainstorming. We worked on the challenge of developing a CRM model according to the current hotel needs, identifying the features they considered most important. The result of the session was the identification of several needs that should be prioritized above others. The huge majority of the attendees agreed that they can not easily identify, segment and categorize their customers by their preferences or consumption habits. A need to improve customer data available was detected, both to increase knowledge of hosted and returning customers and those who have not yet been brand customers. Other needs identified were: manage experiences, know the level of customer loyalty, plan customer loyalty and plan campaigns and follow-ups. Most of the attendees agreeded that knowledge of the communication channels used by their guests and their activity in social media, before, during and after their trip, was critical. In this regard, the hotel can improve the traveler’s experiences, as well as being able to maintain fluid communication with them in all steps of the customer journey.
- *CRM literature review*: In Sect. 2 some of the main theoretical concepts existing in the CRM domain are presented. We used most of those concepts in the development of our ontology.

#### Reengineering of Non-Ontological Resources

We decided to carry out only the first step proposed by scenario 2 of NeON methodology since our ontology combines contributions from ontological resources and steps two and three of NeON are designed to obtain a final ontology from non-ontological resources only. Therefore, the selected aspects of the non-ontological resources will be incorporated into the proposed ontology at the time of the restructuring step along with the ontological resources:

#### Step 1 – Reverse Engineering

**Fig. 7:**
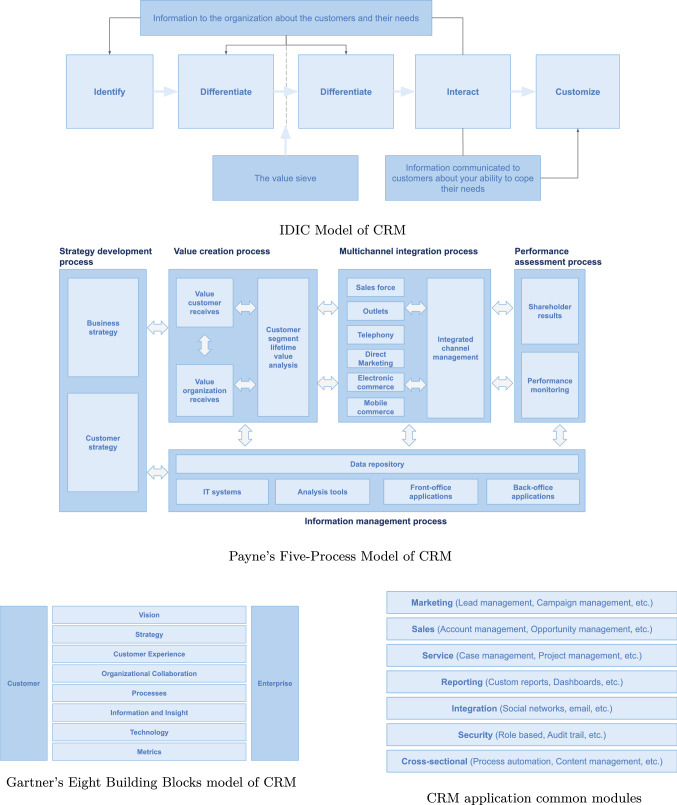
Components of non-ontological resources

The main goal of this activity is to analyze the selected non-ontological resources and identify their underlying components and then create representations of the resources at different levels of abstraction. Figure 7 shows the schemes of the following resources:

- *IDIC*: From this model, we take into account the four steps that it proposes to assess customers’ expectation and their value to the business: identify customer expectations, differentiate customer expectations, customer interactions and customizations to meet customer expectations (Fig. 7a).
- *Payne’s Five-Process*: We consider the processes it proposes to help building and maintaining relationships with customers: strategy development process, value creation process, multichannel integration process, performance assessment process, and information management or analytical process in CRM (Fig. 7b).
- *Gartner’s Eight Building Blocks model*: We consider the 8 building blocks proposed by Gartner to be successful in CRM, vision, strategy, customer experience, organizational collaboration, processes, information and insight, technology and metrics (Fig. 7c).
- *CRM application common modules*: From the analysis carried out of the current and future features of various commercial CRM applications, more than 100 concepts have been identified, grouped into marketing, sales, services, reporting, integration, security and cross-sectional categories, that we have taken into account in our model (Fig. 7d).
- *CRM main concepts in hospitality industry*: From the focus group and the interviews, we reviewed and obtained insights on the concepts and their importance in this sector, highlighting Buyer Persona, customer data, customer experiences, customer loyalty and campaign management.
- *Other non-ontological resources*: In our review of the scientific literature, different concepts were found that are not included in the previous sources, such as, customer relationship stages, buyer persona, customer journey and flywheel.

#### Reuse of Ontological Resources

Applying scenario 4 of the NeOn methodology (reusing and reengineering ontological resources), we searched for ontologies taking the requirements of Sect. 4.2.1 as a reference. The initial search result for domain-specific CRM ontologies was poor so we decided to expand it and include other ontologies related to some important aspects of CRM such as the customer or business models. The CRM related ontologies analyzed were:

- *Customer Ontology* (Changrui and Yan 2011): this ontology focuses on analyzing the customers of a company, identifying generic market concepts, types of customers, activities carried out by customers and customer statuses.
- *Customer Personality Ontology* (Ramadhanti et al. 2020): this ontology, based on the Big Five Personality theory, proposes modeling the personality of a customer in order to help the company define the most appropriate relationship strategy. The categories of principal intereset are openness, conscience, extraversion, agreeableness and neuroticism.
- *Business Model Ontology* (Osterwalder 2004): in this case, the ontology proposes modeling a business based on nine blocks related to different parts of the company. One part, the customer interface covers all customer related aspects. This comprises the choice of a firm’s target customers, the channels through which it contacts them and the kind of relationships the company wants to establish with its customers. The customer interface describes how and to whom it delivers its value proposition, which is the firm’s bundle of products and services.
- *CRM ontology based on CMMI *(Lee et al. 2007): a CRM Ontology it is proposed based on CMMI project planning for business application. The CRM ontology describes the CRM project planning knowledge based on CMMI. It contains three categories, namely, establishing CRM project estimates, developing a CRM project plan, and obtaining CRM project commitment.
- *CRM Ontology based on DOLCE* (Magro and Goy 2012): the main contribution of this ontology is the proposal of O-CREAM-v2, a core reference ontology for the CRM domain, specifically targeted to Small and Medium Sized Enterprises (SME). The design of O-CREAM-v2 has been based on requirements mainly elicited from domain analysis, and has been developed within the framework provided by the well-known DOLCE foundational ontology, together with three DOLCE extensions. O-CREAM-v2 is structured into five modules, namely, Relationships, Knowledge, Activities (all three spanning from the upper and the lower core), Software and Miscellaneous (both limited to the upper core).

For our ontology, we finally selected BMO and O-CREAM-v2, those that we identified closest to our requirements and that had a certain level of development.

#### Reengineering of Ontological Resources

For each of the selected ontologies, we first performed the reverse engineering step. Next, in the restructuring step, we merged the selected ontological concepts with the non-ontological ones, all of them based on the foundational UFO ontology.

#### Step 1 – Reverse Engineering

**Fig. 8:**
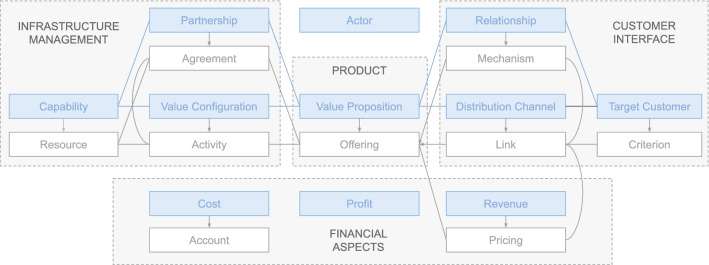
The business model ontology
- *O-CREAM-v2*: No code implementations of this ontology were found but it is well described and its conceptual model is clearly stated in Magro and Goy (2012). It is based on the founding ontology of DOLCE, the ontology of Descriptions and Situations (DnS), the ontology of information (OIO) and the Ontologies of Plans (OoP). O-CREAM-v2 is currently structured in five modules: relationships, knowledge, activities, software and miscellaneous modules, as is illustrated in Fig. 9. The Relationships module provides a characterization for a basic notion of relationship and for the main kinds of business relationships in the CRM domain. They distinguish two levels, upper core and lower core. The latter is more specific to the considered CRM domain, while the former is more general, but can also be useful in business domains other than CRM.

The main goal of this activity is to output a possible conceptual model on the basis of the code in which the ontological resources are implemented:

- *BMO*: Although several OWL versions were found with the implementation code of this ontology, its direct processing was not necessary since there is documentation about the conceptual model that this ontology represents. The business model ontology is a set of elements, and their inter-relationships aim at describing the money earning logic of a firm. The ontology contains nine business model building blocks, so-called business model elements, and is illustrated in Fig. 8.

**Fig. 9:**
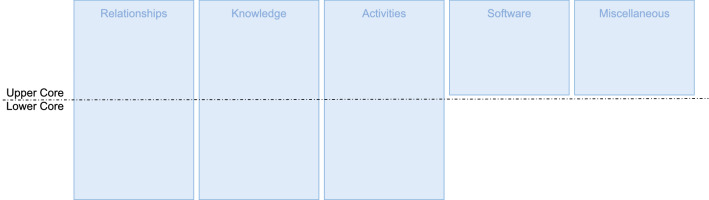
O-CREAM-v2 modules and levels

**Table 3:** CURIE-O Ontology Classes and Origin

CURIE-O Class	Concept or related class	Knowledge resource
Strategy	Business strategy, customer strategy	Payne’s five-process
ValueProposition	Value proposition	Business model ontology
BuyerPersona	Target customer	Business model ontology
Channel	Distribution channel	Business model ontology
CustomerRelationshipStage	Relationship	Business model ontology and O-CREAM
CustomerRelationshipActivity	Activity	O-CREAM
InformationElement	Information element	O-CREAM
Organization	Actor	Business Model Ontology
CustomerJourneyStage	Customer journey stages	Vázquez et al. (2014)
CustomerJourneyActivitie	Customer journey activities	Lemon and Verhoef (2016)
CustomerJourneyExperience	Customer journey experiences	Lemon and Verhoef (2016)
Analytic	Metrics	Gartner’s eight building blocks model

#### Step 2 – Restructuring

The main goal of this activity is to correct and reorganize the knowledge contained in the conceptual models of the ontological resources and detect missing knowledge. Since we only decided to select certain aspects of the domain ontologies, we did not start from by pruning the original ontology. Instead, we decided to create a new ontology where we regroup the elements of interest according to the requirements:

- *BMO*: From this ontology, we selected the Product and Customer Interface pillars. This will help understand the essence of and the relation between a company’s value proposition, target customer segments, distribution channels and the actual customer interactions: (1) Value Proposition: this is an overall view of a company’s bundle of products and services that are of value to the customer; (2) Target Customer: this is a segment of customers a company wants to offer value to; (3) Distribution Channel: this is a means of getting in touch with the customer; (4) Relationship: this describes the kind of link a company establishes between itself and the customer; (5) Actor: this is an employee, someone external or an organization that executes activities in the context of the company.
- *O-CREAM*: From this ontology, we selected the Relationships, Knowledge and Activities modules for their fit with the requirements of our ontology. Inside these modules we chose the following concepts: (1) Relationship: many states of affairs in the CRM domain can be conveniently described as aggregations of interrelated particulars, in which each particular plays (one or more) specific roles. Such aggregations are defined as relationships. For instance, when an enterprise sells a person goods, a particular business relationship is established between the enterprise that is selling and the person that is buying. Moreover, such a relationship also involves other particulars, such as the goods sold and, possibly, other elements such as the specification of payment and delivery methods, etc. This ”relationship”, as intended in the CRM domain, can be effectively modeled as a ”relationship” in the formal sense; (2) Information Element: the CRM activities manage a huge amount of business knowledge, they access the master data files, the transactional data and various information sources, they analyze data and information, they produce data, documents and reports, they register new contacts in databases, orders, sales, communications, etc; (3) Activity: the notion of activity plays a central role. This is a rather broad concept that encompasses, among other things, the core business activities in the CRM domain (e.g., acquiring a new contact, selling a product, making an offer, communicating with a customer, etc.); the activities related to information management (e.g., reporting, handling documents, performing a statistical analysis on sales, clustering customers into different groups sharing similar characteristics, etc.); the specific activities supporting the main processes (e.g., creating a bill, printing an invoice, changing the format of an electronic document, etc.). Each activity always has a starting time and can have an ending time, too; it can receive some particulars as inputs and involve some particulars as outputs; it is always executed by someone or something; it can exploit some resources and it may be performed according to some specified methods.

**Table 4:** Selected Classes and UFO Stereotype Correspondence

CURIE-O Class	UFO stereotype
Strategy	Event
ValueProposition	Relator
BuyerPersona	Kind
Channel	kind
CustomerRelationshipStage	Relator
CustomerRelationshipActivity	Event
InformationElement	Kind
Organization	Kind
CustomerJourneyStage	Phase
CustomerJourneyActivity	Event
CustomerJourneyExperience	Mode
Analytic	Event

As already discussed, we must add to this list of selected ontological classes those concepts chosen from non-ontological resources. The result is shown in Table 3, where we see CURIE-O classes and its ontology class or non-ontological concept origin.

#### Step 3 – Forward Engineering

The main goal of this activity is to output a new implementation of an ontology on the basis of the new conceptual model. We finally decided to implement CURIE-O by using OntoUML. OntoUML addresses many problems in conceptual modeling and has been successfully applied in different contexts (Pergl et al. 2013). We performed an ontological analysis of this selected knowledge resources based on UFO in order to reveal the correspondences between the knowledge resources elements and concepts in the foundational ontology. This allows the adjustment of previous knowledge resources, or portions of them, for integration into the proposed UFO-based ontology (see Table 4).

**Fig. 10:**
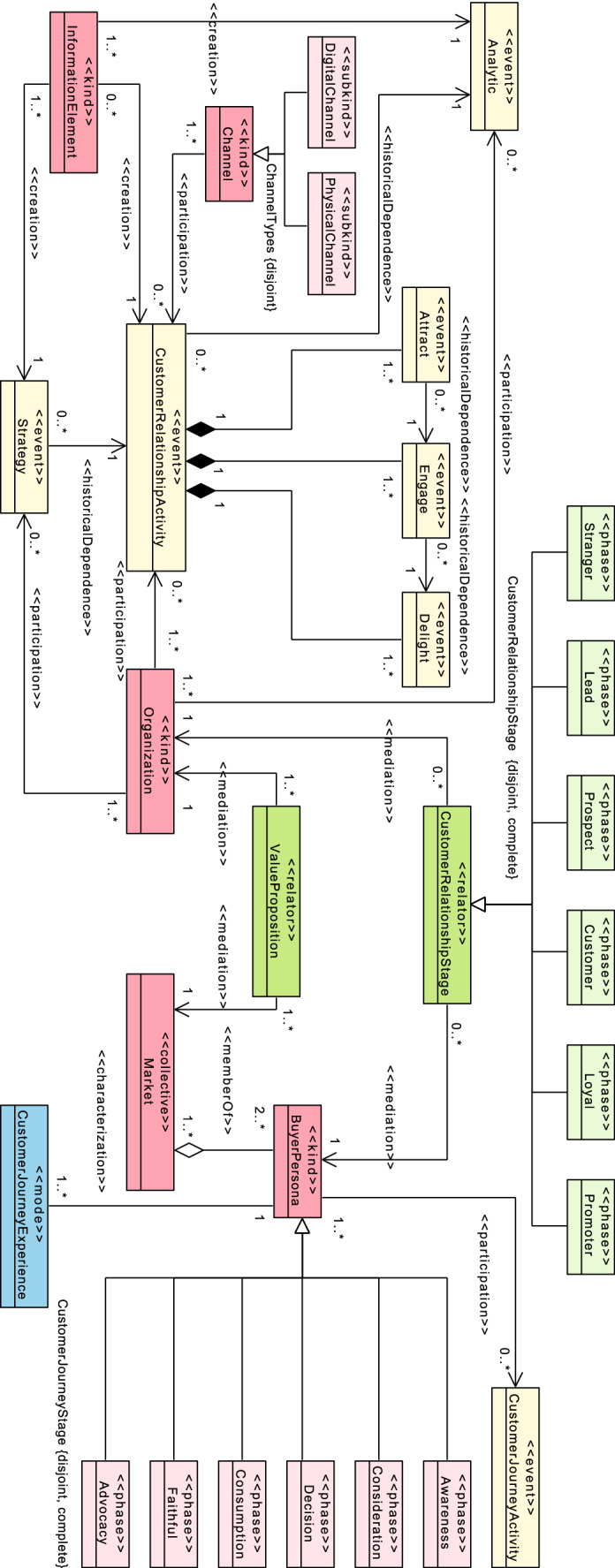
CURIE-O ontology in OntoUML language

From these selected classes and the mapping carried out to UFO concepts, in Fig. 10 we show the CURIE-O ontology described in the OntoUML modeling language. The ontology is made up of 29 classes around the 12 concepts selected in the construction process.

CURIE-O is briefly described below, grouped into the 12 main components:

- *Strategy*:
  - OntoUML stereotype:<<event>>
  - Description: it indicates, through the definition of plans, how to achieve the purpose and vision. One of the main activities of the Strategy is to identify the BuyerPersonas of the company, understanding their needs and their expected experiences.
  - Relations:<<participation>> of Organization,<<creation>> of InformationElement and<<historicalDependence>> of CustomerRelationshipActivity.
- *ValueProposition*:
  - OntoUML stereotype:<<relator>>
  - Description: this is what the company offers to the Market, in particular to its BuyerPersona, to solve or satisfy any of their needs.
  - Relations:<<mediation>> between Organization and Market.
- *BuyerPersona*:
  - OntoUML stereotype:<<kind>>
  - Description: this is a customer profile, a semi-fictional representation of the ideal customer based on information such as demographics, behaviors or motivations.
  - Relations: ValueProposition<<mediation>> with Organization,<<participation>> in CustomerJourneyActivity, a<<memberOf>> Market, a<<characterization>> of CustomerJourneyExperience and it can be in different CustomerJourneyStage (Awareness, Consideration, Decision, Consumption, Faithful, Advocacy).
- *Channel*:
  - OntoUML stereotype:<<kind>>
  - Description: it describes how the company manages to get its value proposition to the customer, both physically and at the communication level. These channels can be physical or digital.
  - Relations:<<participation>> in some of the execution of CustomerRelationshipActivity and it is divided into PyshicalChannel and DigitalChannel.
- *CustomerRelationshipStage*:
  - OntoUML stereotype:<<relator>>
  - Description: it describes the stages through which a BuyerPersona can pass in their relationship with a company.
  - Relations:<<mediation>> between Organization and BuyerPersona and the stages can be divided into Stanger, Lead, Prospect, Customer, Loyal, Promoter.
- *CustomerRelationshipActivity*:
  - OntoUML stereotype:<<event>>
  - Description: it describes the activities that an Organization carries out in its relationship with customers or potential customers.
  - Relations: Organization<<participation>> in carrying out the activities, Channel<<participation>> in activities, InformationElement and Metric can be<<creation>> and Attract, Engage and Delight are part of the activities, and<<historicalDependence>> of Analytic
- *InformationElement*:
  - OntoUML stereotype:<<kind>>
  - Description: this describes the business knowledge that is managed in CustomerRelationshipActivity, such as documents, registers or reports.
  - Relations: CustomerRelationshipActivity and Strategy<<creation>> information.
- *Organization*:
  - OntoUML stereotype:<<kind>>
  - Description: this is the company, a department or an employee who executes activities in the context of the organization.
  - Relations:<<mediation>> in CustomerRelationshipStage and in ValueProposition,<<participation>> in the development of Strategy and the activities of CustomerRelationshipActivity
- *CustomerJourneyStage*:
  - OntoUML stereotype:<<phase>>
  - Description: this describes the stages through which a BuyerPersona can be in its customer journey.
  - Relations: the stages are Awareness, Consideration, Decision, Consumption, Faithful, Advocacy.
- *CustomerJourneyActivity*:
  - OntoUML stereotype:<<event>>
  - Description: this describes the activities that a BuyerPersona carries out in its customer journey.
  - Relations: BuyerPersona<<participation>> in carrying out the activities
- *CustomerJourneyExperience*:
  - OntoUML stereotype:<<mode>>
  - Description: this describes the experiences lived by BuyerPersonas during their customer journey in each of the points of contact with the organization
  - Relations: this is a<<characterization>> of BuyerPersona
- *Analytic*:
  - OntoUML stereotype:<<event>>
  - Description: the analysis of performance indicators of activities are calculated by analytical techniques (see Sect. 2.3)
  - Relations:<<participation>> of Organization,<<creation>> of InformationElement and<<historicalDependence>> of CustomerRelationshipActivity

### Demonstration and Evaluation

According to March and Smith (1995), by building instantiations we operationalize constructs, models and methods they contain, thus demonstrating their feasibility and effectiveness. Furthermore, by evaluating instantiations we also provide confirmation for underlying artifacts.

Therefore, to demonstrate and evaluate our ontological model, we have developed an instantiation of it in the application prototype shown in Sect. 6.

## Artifact 2: EA-Based CRM Model

### Problems, Motivation and Objectives of the Solution

**Table 5:** CURIE-EA architecture principles

Principle	Statement	Rationale	Implications
Buyer Persona and Customer Journey	Every company must identify who its ideal customers are, what characteristics they have, and what journey they follow in their purchasing process	In order to optimize the finite resources of a company, you must focus on those customers that you can really satisfy and with whom you can achieve a greater profit margin	Identify buyer persona, make a detailed profile and communicate to the entire organization who they are and how to treat them
Value proposition	Every company must clearly define its value proposition	In order to attract clients and compete in the market, every company must define, design and communicate what it sells and what makes it different	Analyze the needs of the buyer person and analyze competitors to design an appropriate value proposition
Customer relationship process	Every company must implement in its organization all the processes of relationship with its clients	A client will not be satisfied if they do not have an adequate experience in each of the interactions they have with the company	Design each process to be able to fulfill all relationships with clients and train the personnel involved
Channels	Every company must identify, select and use those interaction channels in which its clients are	If we want customers, we must go find them in the channels where they are	Identify our clients’ channels and prepare the organization to interact through these channels
Customer 360 and data accessibility	All data related to customers must be registered and accessible by all those in the organization who need them	The customer global and unique vision will allow us to know them better in order to satisfy them	Educate organization so they understand the relationship between value of customer data, sharing it, and accessibility to it
Data security	All data relating to customers must be managed in accordance with current legislation and with the necessary security	There is legislation in most countries that requires information to be managed in a specific way and applications must apply it	Know the current legislation and adjust applications and procedures to comply with it
Technology independence	Applications are independent of specific technology choices and therefore can operate on a variety of technology platforms	Independence of applications from the underlying technology allows applications to be developed and operated in a most cost-effective and timely way	Select and implement standards that support portability
Ease-of-use	Applications are easy to use. The underlying technology is transparent to users, so they can concentrate on tasks at hand	It is a positive incentive for use of applications and increase productivity. Most of the knowledge required to operate one application will be similar to others and training is kept to a minimum	Define and apply a design system so that the applications have the same “look and feel” and share the same ergonomic requirements
Microservices architecture	As a way to comply with “Technology independence” principle, the architecture will be oriented towards microservices	This way of designing applications allows independent technological decisions for each microservice and allows to evolve in a decoupled way	Design applications by decomposing them into independent problems with delimited contexts
Containers architecture	For scaling, updating and maintenance reasons, the technological architecture will be container-based	Containers are the type of technology usually chosen to support a microservices-oriented architecture	Choose and implement specific container creation and orchestration technology

#### Problems and Motivation

From the perspective of research in information technology, much emphasis has been given to the Enterprise Architecture (EA) discipline with respect to enterprise management (Lankhorst 2013). In this regard, the Open Group develops and maintains the TOGAF specification for developing enterprise architectures (The Open Group 2018). It defines a foundational structure used for developing several architectures. It covers from business processes to technological and infrastructure aspects. The context of the problem and the motivation is the same as indicated in Sect. 4.1. However, the goal of the solution provided with this second artifact is to complement the first one. While an ontology-based model provides an unifying framework, it helps to share and to reuse knowledge, and facilitates communication within a domain, an EA model unequivocally describes, analyzes, and visualizes how an organization should operate from a business, application, and technology perspective.

#### Objectives of the Solution

We want to design CURIE-EA, an EA-based CRM model that enables the expression of the CRM process as well as the expression of its relationship with the operational aspects of an enterprise’s architecture. We want to use ArchiMate as our modeling language (The Open Group 2019) and describe it from the Business, Application and Technology perspectives.

Our goal is that CURIE-EA could be considered as an EA-based CRM reference model, particularly in the business and application layers, as they are more generic layers modeled using the concepts and relationships of CURIE-O. On the other hand, the technology layer will propose a microservices-based architecture and the use of a set of technologies (development languages, databases and operating systems) as a possible implementation option, but other technologies could also be valid.

Architecture principles define the underlying general rules and guidelines for the use and deployment of IT resources and assets across the enterprise. They reflect a level of consensus among the various elements of the enterprise, and form the basis for making future IT decisions (Greefhorst and Proper 2011; Haki and Legner 2021). Based on the information collected in our research, our model must comply with the architecture principles shown in Table 5.

These principles are ordered and related to the different layers of the TOGAF model: (1) Business layer: Buyer Persona and Customer Journey, Value proposition, Customer relationship process, Channels and Customer 360 and data accessibility; (2) Application layer: Data security, Technology independence and Ease-of-use; (3) Technology layer: Microservices architecture and Containers architecture.

### Design and Development

**Fig. 11:**
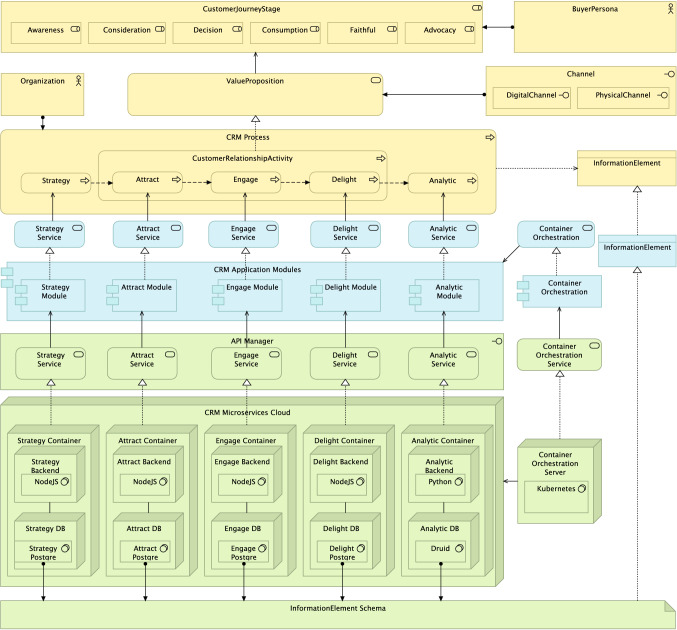
CURIE-EA, an enterprise architecture CRM model
- *Application layer*: To carry out the activities mentioned in the business layer, those of the CRM process, the Application layer offers a set of services that are supported by the modules of Strategy, Attract, Engage, Delight and Analytic. These modules are in turn grouped into other modules that we have decided not to include in this model for the sake of simplicity. Some of these modules are shown in the discussion of non-ontological resources presented in Sect. 4.2: lead management, campaign management, account management, opportunity management, case management, social networks integration, etc. We have modeled an application for each process of the business layer since our proposal is based on the microservices architecture. The Container Orchestration application is one of the essential applications that a microservices architecture must have, so we have incorporated this application into our model to reflect the microservices proposal.
- *Technology layer*: For the design of the Technology layer we have decided to organize it according to the microservices approach. The popularity of microservice-based architectures is increasing in many organizations since this new software architecture style supports the continuous delivery approach by releasing the rigid structure of monolithic systems towards independent deployments of single applications (Song et al. 2019). Each of the applications of the previous layer has an associated service that will run in a container as a microservice, having its own business logic and its own database decoupled from all other services. Although not reflected in the model, all these microservices’ communication interfaces can interact with one another, offering the final functionality demanded by the Application and Business layer. As an example of technologies, we mention the use of Kubernetes for the container orchestration server part, Node.js or Python for the backend microservices part, and PostgreSQL and Druid for the microservices databases. For simplicity, other elements of the microservices architecture have not been included, such as: IAM, Service Mesh, API gateway, etc. (Pinheiro et al. 2019). Microservices have created more adaptable and flexible IT infrastructures. Each of the services can be deployed and modified without affecting other services or functional aspects of the application.

The design of this second artifact is based on CURIE-O, our ontological model presented in Sect. 4. The chosen framework has been TOGAF along with the ArchiMate modeling language. The layers presented are those of Business, Applications and Technology. The tool used is the Archi modeling toolkit. The proposed model is shown in Fig. 11. This model consists of three layers:

- *Business layer*: Our BuyerPersona is a Business Actor referring to the customers and leads that a company may have. The different steps which a customer goes through in their customer journey and their relationship with a company, CustomerJourneyStage, are modeled as a Business Role. Each stage of the customer journey (Awareness, Consideration, Decision, Consumption, Faithful and Advocacy) can be seen as different ”roles” that a customer adopts demanding a different service from the company. These services and products that the company offers are included in the ValueProposition that we model as a Business Service, whereas the services are carried out through Channels modeled as a Business Interface. In the model we show the existence of a DigitalChannel and a PhysicalChannel. In order to get the ValueProposition to the BuyerPersona, the company carries out a set of activities, Strategy, CustomerRelationshipActivity and Analytic, all of them represented as a Business Process. The first of these activities, Strategy, is carried out by the company seeking to define the way in which it will manage its customers. Next, the CustomerRelationshipActivity activity is designed to execute this strategy, seeking Attract, Engage and Delight customers and potential leads. The company wants to draw their attention, interact with them, solve their problems until they become customers. Finally, they want customers to achieve loyalty so that they continue to buy and recommend the products and services of the company. The next activity is that of Analytic, an activity that seeks to measure the entire previous process to know what is working properly and adjust the strategy, where necessary. All these activities are carried out by the Organization, modeled as a Business Actor. Obviously, this entire process uses and requires information, InformationElement modeled with a Business Object, in order to correctly carry out the activities: contact data, customer journey stage, relationship status (Stranger, Lead, Prospect, Customer, Loyal, Promoter), etc.

We state that the CURIE-EA model proposed has a level of detail that we consider sufficient to show the logic of the CRM model and, at the same time, represent the knowledge obtained from the CURIE-O ontology

### Demonstration and Evaluation

As already stated in the demonstration and evaluation of the ontological model, we will follow the same process for the EA model. Again, according to March and Smith (1995), we will create an instantiation based on CURIE-EA architecture that, when evaluated, will confirm the feasibility and effectiveness of the EA model. The instantiation proposed is the same Task Manager application prototype shown in Sect. 6.

## Artifact 3: Application Prototype Based on our CRM Model

### Problems, Motivation, and Objectives of the Solution

#### Problems and Motivation

The problem to be solved with this third artifact is to confirm whether a real application can be developed taking as a reference the models developed with artifacts 1 and 2.

Although one of our ultimate goals is to develop a complete CRM prototype based on these conceptual models, we wanted to carry out a first validation of a single part of the model.

For this first prototype, it was decided to develop a task manager because it is transversal to the entire CRM process and because it includes several of the concepts and relationships identified in the conceptual model.

#### Objectives of the Solution

The objective of the application is to offer an environment so that the users and teams involved in the CRM process can collect and process their tasks, schedule their execution and keep track of their execution status. This application will have a web architecture made up of the following components:

- Frontend, to implement the user interface.
- Backend, to implement the main logic of the process and the interaction with the database.
- Database, to implement the entities and process relationships.

### Design and Development

Following the guidelines of the DSR method, and after several iterations between our initial CRM conceptual models and the prototype, we reached the design of the final prototype presented in this section.

Some of the lessons learned when implementing the prototype were:

- Observe that certain concepts of the ontological model may need to be decomposed to cover the use cases of the real context of the problem. For example, in the ontology we identified the concept CustomerRelationshipActivity and with the prototype we broke it down into Task, Subtask, Projects and Processes. We did not apply this information to the ontology, but it could be a way to evolve it.
- Identify that certain processes were not reflected in the EA processes. For example, the Analytic process was identified, but not implemented, by the users of the prototype and it was decided to add it to both the enterprise architecture and the ontology.
- Learn which specific technologies to satisfy the architecture principles and requirements of the EA model. Technologies such as Kubernetes, Docker, Node.js or PostgreSQL were tested and, given the positive results, they were incorporated into CURIE-EA as examples of valid technologies.

The methodology used for prototype development was Scrum (Sutherland and Coplien 2019). For the initial requirements definition (identification of actors and user stories), we used as a reference our ontology-based and EA-based models.

From CURIE-O, we used the concepts of CustomerRelationshipActivity, InformationElement, Organization, and BuyerPersona. The relationship between these ontological concepts and the entities defined in the prototype database is as follows:

- *Customer relationship activity*: Task, subtask, project and process
- *Information element*: Document
- *Organization*: User, team
- *Buyer Person*: Client

From CURIE-EA, we used different components of its layers:

- *From the business laye*r: BuyerPersona and Organization business actors, InformationElement business objects and CRM Process business process. The process chosen for the prototype, the Task Manager process, would not be reflected as such in CURIE-EA since it is a lower-level process that would be part of the current processes already shown in the model.
- *From the application layer*: As we discussed in the Business layer, the service and module associated with the Task Manager process would be included in the services and modules currently shown in CURIE-EA.
- *From the technology layer*: We implement the Container Orchestration Server through Kubernetes, we deploy the Task management application in a Docker container, we develop the Backend with Node.js, and we model the database with PostgreSQL.

From this initial requirements definition, the objectives or week sprints were set to develop the prototype. The tools and technologies used were the following:

- *For user interface design*: www.figma.com. It is an open source web tool for visual prototype design. In addition, and in order to unify and accelerate the design of the user interfaces, we decided to use the Ant-design system, developed and maintained by Ant Financial, a company created by Aliplay (Alibaba Group) with the aim of having design and development foundations for all its digital products.
- *For data model design*: PostgreSQL. It is an open source relational database that allows us to implement the entities, fields and relationships extracted from the conceptual model.
- *For backend layout*: Node.js. It is a multiplatform, open source execution environment for the server layer based on the Javascript programming language with an event-oriented architecture and related to Google’s V8 engine.
- *For frontend layout*: React. It is an open source Javascript library designed to create user interfaces with the aim of facilitating the development of applications on a single page. It is maintained by Facebook and the free software community.

#### Database Model

**Fig. 12:**
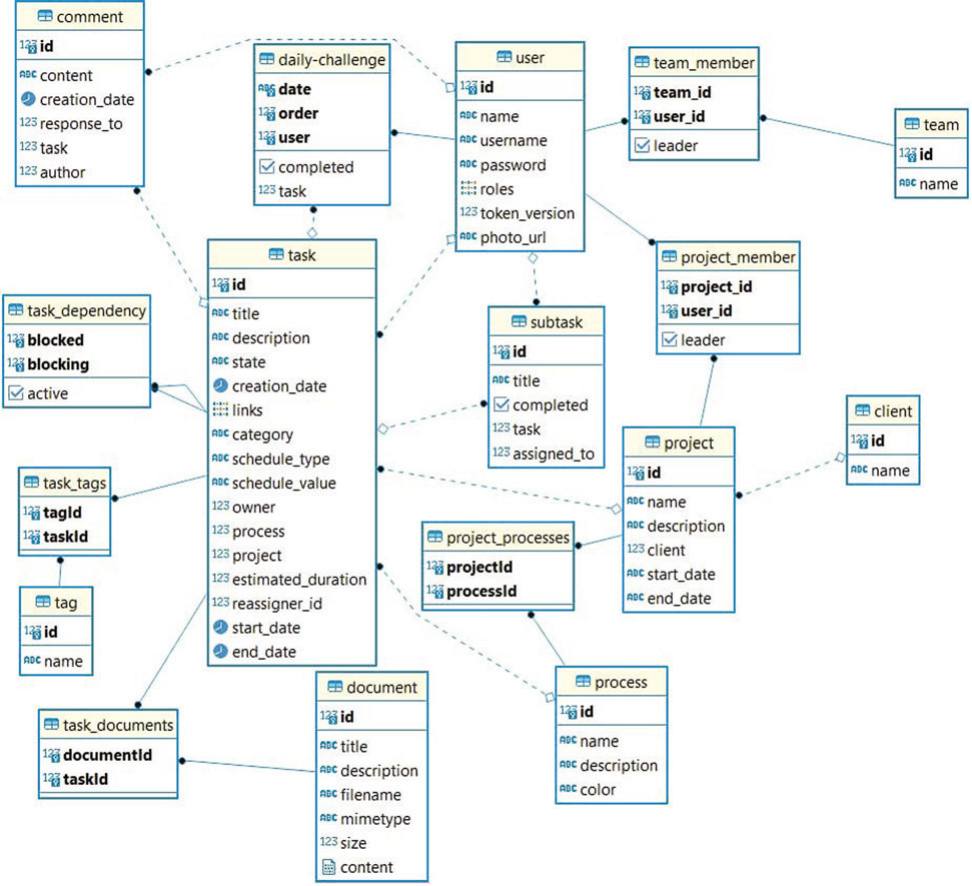
Relational model of the task manager prototype

The data model designed is shown in Fig. 12. The main entities implemented in this Relational Model, that are part of the conceptual model, are: task, subtask, project, process, document, user, team and client.

#### Task Manager Prototype

**Fig. 13:**
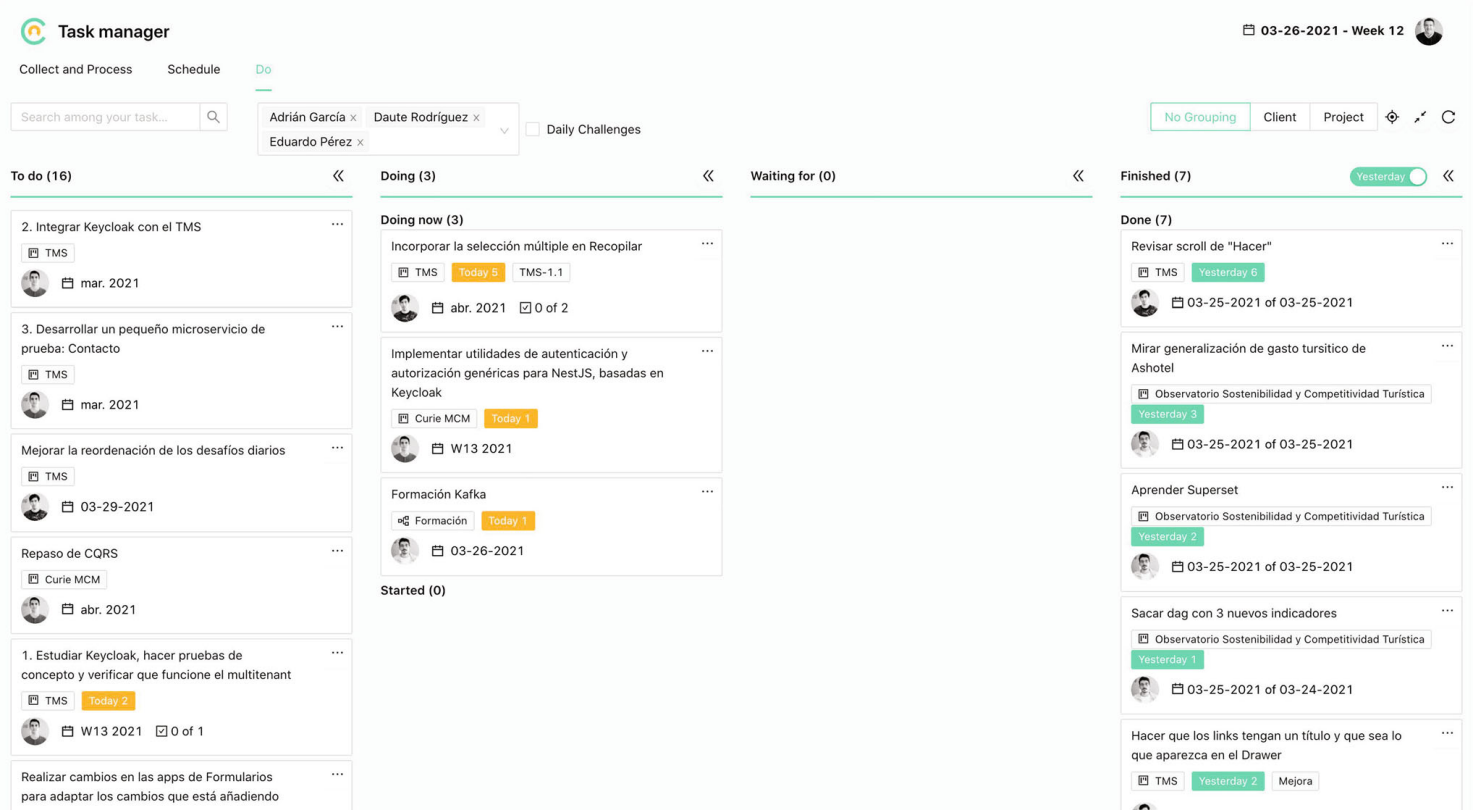
To-do step screenshot of the task manager prototype

The prototype application finally designed is shown in Fig. 13. Although this screenshot refers to the To-Do step, the task manager is organized in the following three steps that propose to organize the tasks of the CRM process as follows:

- *Collect and Process step*: In this stage of the task management, the aim is for the user to be able to collect all those tasks that they have to perform. In addition, they can process and classify them quickly by carrying out a drag and drop.
- *Schedule step*: This next stage is where the tasks that must be performed separately are found, and they may also be dragged and dropped to indicate if they should be performed on a specific day, week or month.
- *To-do step*: This last stage shows the columns of To-Do, Doing, Waiting for and Done, that will allow a simple view of the tasks assigned and their current state. It also allows daily challenges to be set.

### Demonstration and Evaluation

According to Mijač (2019), the method selected for the evaluation of our instantiation is a survey of the application users in which they are asked to evaluate a set of properties, commonly used in DSR for the evaluation of instantiation-type artifacts.

The criteria chosen and the questions asked were:

- *Efficacy*: The degree to which the artifact achieves its goal, considered narrowly, without addressing situational concerns.
- *Usefulness*: The degree to which the artifact positively impacts the task performance of individuals.
- *Technical feasibility*: Evaluates, from a technical point of view, the ease with which a proposed artifact could be built and operated.
- *Accuracy*: The degree of agreement between outputs of the artifact and the expected outputs.
- *Performance*: The degree to which the artifact accomplishes its functions within given constraints of time or space.
- *Effectiveness*: The degree to which the artifact achieves its goal in a real situation.
- *Ease of use*: The degree to which the use of the artifact by individuals is free of effort.
- *Robustness*: The ability of the artifact to handle invalid inputs or stressful environmental conditions.
- *Scalability*: The ability of the artifact to either handle growing amounts of work in a graceful manner, or to be readily enlarged.
- *Operational feasibility*: Evaluates the degree to which management, employees, and other stakeholders, will support the proposed artifact, operate it, and integrate it into their daily practice.
- *Utility*: Measures the value of achieving the artifact’s goal, i.e. the difference between the worth of achieving this goal and the price paid for achieving it.
- *Validity*: It means that the artifact works correctly, i.e. achieves its goal accordingly.
- *Completeness*: The degree to which the activity of the artifact contains all necessary elements and relationships between elements.
- *Adaptability*: The ease with which the artifact can work in contexts other than those for which it was specifically designed.
- *Reliability*: The ability of the artifact to function correctly in a given environment during a specified period of time.
- *Simplicity*: The degree to which the structure of the artifact contains the minimal number of elements and relationships between elements.

The survey was carried out with these 16 questions allowing a rating from 1 to 10, with 1 being a very low rating and 10 a very high rating. The survey was conducted with a team of 12 people from the company Itop Management Consulting who had been using the application for more than two months. This company offers technology consulting services and has a very active day-to-day relationship with clients and task execution.

The weight given to each of the questions was the same and the final result of the survey was a valuation of 8.3 out of 10. Therefore, we consider that the prototype solves the problem for which it has been formulated and that the models in which it was inspired, CURIE-O and CURIE-EA, are valid, at least in this scenario.

## Discussion, Implications, and Limitations

As we have stated throughout this article, CRM is a key process for organizations and its complexity has increased considerably mainly due to changes in customers’ buying habits and the number of channels they use. In order to help companies manage this process, we have proposed two new updated CRM conceptual models, based on ontology and an enterprise’s architecture. We have also demonstrated its validity with the implementation of a prototype based on those models.

By designing an ontology-based model, doing it on the basis of a foundational ontology (UFO) and using its associated language (OntoUML), we are providing a model with semantics and standardization that reuses base concepts already modeled and tested.

By designing an EA-based model (TOGAF), using its associated language (ArchiMate) and relying on the concepts and relationships identified in the ontology, we are providing a semantic model with a business perspective in its Business, Application and Technology layers.

Both models can be used to better understand the existing concepts and relationships in CRM as well as a reference in the design, development and maintenance of technological solutions in this domain.

Although a real prototype has been developed based on these models, it has been only implemented for the management of activities, so there could be a limitation when prototyping other CRM subprocesses. Our intention is to continue developing new prototypes and to validate the proposed models with other subprocesses. Following the DSR methodology, along with the experience of these new prototypes, the necessary adjustments will be made in the models iteratively until their validity is confirmed.

Another limitation of our model could be in the level of detail of the concepts and relationships used, which could be insufficient, depending on the intended use. It has already been mentioned in Sect. 6.2 that, in the prototype implementation, we worked with higher level concepts than those identified in the ontology.

It must also be considered that, although in our research process we collected information from the hotel sector, our models are general and designed for any business. This means that concepts and relationships particular to other sectors may not have been considered. In the same way, there are businesses that sell directly to consumers, and others that sell to companies, this could also be a limitation of our proposal by not taking into account specific aspects of one or the other type of business.

Developing the single subprocess prototype does not imply that the ontological model and EA are incorrect, it simply has not been fully validated by developing prototypes that include all concepts and relationships. However, both models comply by answering competency questions in the ontological model, and architecture principles in the EA model. Regarding the limitation of the level of detail of the models, this should not be a problem, since we understand that these details would form part of the concepts and relationships already provided by CURIE-O and CURIE-EA. The number and scope of our models we believe is sufficient since it is part of a comprehensive analysis of many other models and other sources of information. Regarding the industry and business type limitations, we also consider that they would be specific details that would form part of the concepts and relationships that our models already provide. This circumstance was validated by gathering information specific to the tourism sector and working on the prototype with a consulting company. Two different sectors and with two different business models.

## Conclusions and Future Research

Throughout our research, it has been observed how customer behavior and needs are in continuous evolution, mainly due to digitization. The pace of these changes has been especially accelerated because of the situation generated by the recent COVID-19 pandemic. Customers are now more demanding and less loyal if they are not taken care of. Relationships have become more complex, multi-channel and the customer experience has become a key factor to achieving loyalty.

In this situation, companies cannot stand by, and they should adapt to the same rate at which their customers are changing. If not, they could lose them as clients and, thereby, put their survival at risk.

To manage this context, companies need to set themselves up with tools and working methods that will allow them to better understand the customer, and manage their relationships and experiences more appropriately.

CURIE, as an ontology and enterprise architecture model of CRM, has been designed with this reality in mind and taking advantage of the technology currently available. An application developed using this model as a reference would help companies fight bettered equipped to compete in this complex market. They would handle information that they likely did not have before about customers’ behavior and the experience they are living. They could adapt their communication, products and services to real customer needs in the channels where they are active, and thus, achieve a satisfied customer who will not only stay, but will become a company’s promoter.

The customer orientation that companies are experiencing is generating an increasing demand for CRM systems adapted to the digital age. This encourages us to continue working in the research line of customer relationship management.

### Contribution to Research and Practice

With the development of the artifacts of the ontological model and the enterprise architecture model, we believe in providing an updated CRM conceptual model for the current behavior change and digital context.

The CRM ontological model offers a very efficient tool to help IT specialists to analyze requirements for developing new applications related to CRM. It can also be used by business managers to understand the blocks that make up a CRM process, sharing and reusing knowledge and facilitating related communication in this domain.

The EA CRM model allows unambiguous description, analysis and visualization of the current CRM process, indicating how a company should manage, in the current context, the relationship with its customers from three different points of view: business, application and technology.

In general, the main contributions of our research work in the modeling part are:

- From the perspective of the model, the most widely employed modeling frameworks have been used:
  - A UFO/OntoUML Ontological-based CRM model
  - A EA CRM model based on TOGAF/ArchiMate, one of the main enterprise architecture standards
- From the perspective of concepts and relationships, the current concepts of CRM domain have been developed and incorporated:
  - The Buyer Persona as a way to segment customers based on their pain points (Kelly et al. 2017)
  - The activities that the client performs when interacting with a company in the so-called customer journey
  - The customer’s stages from customer’s perspective in their relationship with a company
  - The company activities performed when interacting with a client
  - The customer’s stages from company’s perspective in their relationship
  - The distribution and communication channels, and the possible strategies to follow depending on their type
  - The information types, structured and unstructured, that a company handles during the relationship with its clients

Regarding our third artifact, the application prototype for the management of company activities, in addition to being an evaluation part of the proposed CRM model, is a starting point for the development of the complete prototype. This development has also helped us to select and test the most used frontend, backend and database development technologies for the final application. We have also implemented some of the microservices and multitenant technological architecture components. This type of architecture is what many of the great commercial solutions of applications in the SaaS cloud type are counting on, including the leaders of CRM application development.

In general, the main contributions of our research work in the implementation part are:

- From a frontend perspective, several visual components have been designed that can be reused in the development of other system components.
- From the backend perspective, not only reusable components have been designed, but also libraries to speed up development, make it more compact and improve its stability. Another important aspect is that these backends, designed as microservices, can be called by other microservices so as not to have to redo already developed functionalities.
- From the database perspective, a relational model has been designed on a relational database.

### Future Research

As future research, we should keep validating the proposed model by developing more software prototypes and also a methodology for implementing it in an organization. We also believe that it would be interesting to continue evolving the model to meet customer behaviour changes and eliminate the limitations indicated in previous sections:

- Expand the scope of both models with other aspects such as CRM in specific sectors, CRM for B2B and B2C business models or modeling the personality of customers.
- Develop a complete CRM prototype taking as a reference the complete ontological model as well as the layers of technological architecture proposed in this work.
- Implement the UFO-OntoUML ontological model in a computational ontology such as OWL.
- Perform more ontology and EA validation, aside from instantiation validation.
- Perform more instantiations of other parts of the model.

As a recent example of a new prototype developed based on our CRM conceptual models, we highlight an implementation of the Analytic process that can be consulted at Asociación Hotelera y Extrahotelera de Santa Cruz de Tenerife, ASHOTEL (2021).

## Supplementary Information

Below is the link to the electronic supplementary material.

Supplementary file 1 (pdf 88 KB)
