# Supplementary material for: CURIE: Towards an Ontology and Enterprise Architecture of a CRM Conceptual Model
Source: Bus Inf Syst Eng. 2022 Feb 21;64(5):615–43. doi: 10.1007/s12599-022-00744-0 (PMC8859932; doi:10.1007/s12599-022-00744-0)
Supplement: Supplementary file 1 — (pdf 88 KB) [file 12599_2022_744_MOESM1_ESM.pdf]

CURIE: Towards an Ontology and Enterprise Architecture of a CRM Conceptual Model

Miguel Fernández-Cejas, Carlos J. Pérez-González, José L. Roda-García, Marcos Colebrook

Business & Information Systems Engineering (2022)

Appendix (available online via http://link.springer.com)

Appendix A CURIE: Towards an Ontology and Enterprise Architecture of a CRM Conceptual Model (Supplementary material)

Table 1: Common CRM concepts from 5 CRM comparison. Source: Cruz and Vasconcelos (2015)

Sales	Marketing	Services	Reporting	Integration	Security	Other features
– Account management<br>– Activity management<br>– Competitor tracking<br>– Contact management<br>– Contract management<br>– Lead management<br>– Opportunity management<br>– Product catalog and management<br>– Quote management<br>– Territory management<br>– Quota management<br>– Order management<br>– Sales pipeline<br>– Sales forecasting	– Campaign management<br>– Campaign execution<br>– Email marketing<br>– Marketing campaigns<br>– List management<br>– Web lead to capture<br>– Lead management	– Case escalation and notification<br>– Case routing and queuing<br>– Contact center<br>– Case management<br>– Customer self service portal<br>– Email management<br>– Knowledge base<br>– Customer view<br>– Service analytics<br>– Service contracts	– Custom reports<br>– Dashboards<br>– Sales analytics<br>– Marketing analytics<br>– Service analytics	– Social networks<br>– Email integration<br>– Web-services api<br>– Soap integration<br>– Microsoft office integration<br>– Automatic call distributor<br>– Computer telephony integration<br>– Cloud connectors<br>– Integrated third-party apps	– Role based security<br>– Advanced password management<br>– Control data access<br>– User based security<br>– Team based security<br>– Audit trail	– Workflow and processes automation<br>– Document management<br>– Mobile and offline access<br>– Calendar management

Table 2: Main CRM concepts from 40 CRM comparison. Source: Business-Software (2021)

Marketing	Sales	Services	Other features
– Lead generation<br>– Lead nurturing<br>– Lead scoring<br>– List Segmentation<br>– Campaign management<br>– Community building<br>– Marketing resource<br>– Brand management	– Service catalog<br>– Opportunities management<br>– Competitor tracking<br>– Quotes management<br>– Orders management<br>– Contract management<br>– Invoices management<br>– Billing management<br>– Commissions management	– Project management<br>– Service contracts<br>– Tickets management<br>– Knowledge base	– Account management<br>– Contact management<br>– Customer lifecycle management<br>– Contact center<br>– Task management<br>– Audit trail<br>– Workflow management<br>– Content management<br>– Document management<br>– Omnichannel support<br>– Collaboration tools<br>– Customer satisfaction management<br>– Data Imports<br>– Email management<br>– Social media management<br>– GDPR compliance<br>– Mobile support<br>– Analytics<br>– Security<br>– Integrations<br>– Employee management<br>– Organization management<br>– Forms management

Table 3: Main CRM concepts from Gartner Magic Quadrant. Source: Gartner, Inc. (2021)

CRM Lead Management	Sales Force Automation	Multichannel Marketing Hubs	Voice of the Customer
– Multichannel lead management<br>– Lead aggregation<br>– Lead augmentation and deduplication<br>– Lead process management<br>– Lead nurturing<br>– Lead scoring and qualification<br>– Analytics and measurement<br>– Integration with other applications	– Account and contact management<br>– Activity management<br>– Opportunity management<br>– Guided selling<br>– Forecasting management<br>– Reports and dashboards<br>– Mobile<br>– Partner relationship management<br>– Platform and integration<br>– Lead management	– Customer profile management<br>– Campaign workflow<br>– Event triggering<br>– Personalization<br>– Marketing channel support<br>– Real-time decisions/recommendations<br>– Marketing analytics<br>– Integration with other applications	– Customer feedback collection through multiple channels<br>– Customer direct feedback<br>– Customer indirect feedback<br>– Customer inferred feedback<br>– Customer actionable insight

References

Business-Software (2021) Top 40 customer relationship management software report: Comparison of the leading CRM software vendors. Available at https://www.business-software.com/offer/top-40-crm-software/ (Accessed 27 Dec 2021)

Cruz A, Vasconcelos A (2015) Towards a reference enterprise application architecture for the customer relationship management domain. In: Proceedings of the 17th international conference on enterprise information systems, SCITEPRESS – Science and Technology Publications, Lda., ICEIS 2015, vol 3, pp 185—-195

Gartner, Inc. (2021) Gartner magic quadrant and critical capabilities. Available at https://www.gartner.com/en/research/magic-quadrant-critical-capabilities/ (Accessed 27 Dec 2021)
